# Studies on the molecular level changes and potential resistance mechanism of *Coreius guichenoti* under temperature stimulation

**DOI:** 10.3389/fgene.2022.1015505

**Published:** 2022-10-03

**Authors:** Yuanliang Duan, Qiang Li, Jian Zhou, Han Zhao, Zhongmeng Zhao, Lanmei Wang, Mingkun Luo, Jun Du, Zaijie Dong

**Affiliations:** ^1^ Wuxi Fisheries College, Nanjing Agricultural University, Wuxi, China; ^2^ Freshwater Fisheries Research Center of Chinese Academy of Fishery Sciences, Key Laboratory of Freshwater Fisheries and Germplasm Resources Utilization, Ministry of Agriculture and Rural Affairs, Wuxi, China; ^3^ Fisheries Institute, Sichuan Academy of Agricultural Sciences, Chengdu, China

**Keywords:** *Coreius guichenoti*, transcriptome, proteome, differential expression, temperature stimulation

## Abstract

In this study, we used transcriptome and proteome technology to analyze molecular level changes in tissues of *Coreius guichenoti* cultured at high temperature (HT) and low temperature (LT). We also screened for specific anti-stress genes and proteins and evaluated the relationships between them. We identified 201,803 unigenes and 10,623 proteins. Compared with the normal temperature (NT), 408 genes and 1,204 proteins were up- or down-regulated in brain tissues, respectively, at HT, and the numbers were 8 and 149 at LT. In gill tissues, the numbers were 101 and 1,745 at HT and 27 and 511 at LT. In gill tissues at both temperatures, the degree of down-regulation (average, HT 204.67-fold, LT 443.13-fold) was much greater than that of up-regulation (average, HT 28.69-fold, LT 17.68-fold). The protein expression in brain (average, up 52.67-fold, down 13.54-fold) and gill (average, up 73.02-fold, down 12.92-fold) tissues increased more at HT than at LT. The protein expression in brain (up 3.77-fold, down 4.79-fold) tissues decreased more at LT than at HT, whereas the protein expression in gill (up 8.64-fold, down 4.35-fold) tissues was up-regulated more at LT than at HT. At HT, brain tissues were mainly enriched in pathways related to metabolism and DNA repair; at LT, they were mainly enriched in cancer-related pathways. At both temperatures, gill tissues were mainly enriched in pathways related to cell proliferation, apoptosis, immunity, and inflammation. Additionally, Kyoto Encyclopedia of Genes and Genomes pathway analysis showed more differentially expressed proteins in gill tissues than in brain tissues at HT and LT, and temperature stimulation led to the strengthening of metabolic pathways in both tissues. Of the 96 genes we identified as potentially being highly related to temperature stress (59 from transcriptome and 38 from proteome data), we detected *heat shock protein 70* in both the transcriptome and proteome. Our results improved our understanding of the differential relationship between gene expression and protein expression in *C. guichenoti*. Identifying important temperature stress genes will help lay a foundation for cultivating *C. guichenoti*, and even other fish species, that are resistant to HT or LT.

## 1 Introduction

The cyprinid *Coreius guichenoti* is an economically important fish species that is distributed in the main stream and tributaries of the upper reaches of the Yangtze River, the lower reaches of the Jinsha River, and in the Minjiang River, Jialing River, Wujiang River, and other waterways (Fish laboratory of Hubei institute of aquatic biology, 1976; Ding, 1994). The wild resources of *C. guichenoti* have decreased sharply due to the development of hydropower plants in the Yangtze River Basin (Liu et al., 1990; Yang et al., 2017; Li T. et al., 2020). Its suitable growth water temperature is 20°C–25°C (Yang et al., 2017), but the operation of power station dams greatly affects the water temperature in the river, which in turn has negative effects on fish growth and reproduction (Li T. et al., 2021). By combining body weight and temperature data and the conventional metabolic rate of *C. guichenoti* and establishing a model, Luo and Wang (2012) found that the increased water temperature caused by dam construction in the Yangtze River may lead to a significant increase in the energy demand of this species and have certain ecological consequences.

In 2013, *C. guichenoti* became an incidental species (Liu and Zhou, 2018) and the wild population was listed as a national secondary protected species. As early as 12 December 2007, *C. guichenoti* was included in the list of economic aquatic animal and plant resources under national key protection in China. Compared with the survival of artificial domestication, the artificial cultivation and maturation of parent fish of this species is more difficult (Sun et al., 2020). Therefore, investing in more scientific research is crucial to protecting the population resources of *C. guichenoti*.

Due to the scarcity of natural population resources, studies of *C. guichenoti* now mainly focus on artificial reproduction (Dong et al., 2019; Liu et al., 2020; Liu X. et al., 2021; Hu et al., 2021), domestication (Gao et al., 2012; Qu et al., 2017; Shu et al., 2020), and genetic diversity (Cheng et al., 2015; Xiong et al., 2016; He et al., 2019; Li X. et al., 2021). Studies of meat quality (Xia et al., 2015; Dong et al., 2021), gene transcription (Li X. M. et al., 2020), gene cloning (Li, 2012), and stress response (Zhang and Chen, 2005; Li et al., 2013, 2016; Long et al., 2020) have also been reported.

Fish are affected by various environmental factors in the processes of growth and reproduction. When these environmental factors change, fish undergo various stress reactions. Because of the important economic value of *C. guichenoti* and the scarcity of wild populations, understanding the stress response caused by environmental change has largely attracted scholars’ attention. In the natural environment, parasitic infections can result in erosion of skin and gills, deformation or loss of fin rays, and reduction of relative fatness of *C. guichenoti* (Ran et al., 2005), and the probability of parasitic infection is higher in juveniles than in adults (Ran et al., 2005; Zhang and Chen, 2005). Fu et al. (2019) analyzed the stress response of *C. guichenoti* after infection with *Ichthyophthirius multifiliis* from the aspect of gene expression. Acute handling stress can lead to changes in blood indicators, gill and kidney tissues, and even parasitic infection of *C. guichenoti* (Zhao, 2014; Yuan et al., 2018). Li et al. (2013) reported that only some biological functions in head kidney tissues gradually recovered after 24 h. Using MS-222, reducing temperature, or applying electrical anesthesia could reduce the stress response caused by transportation (Zhao, 2014; Zhao et al., 2014; Zhu et al., 2015; Dong et al., 2017).

Water temperature is an important factor that affects the growth and reproduction of fish, and it has a great impact on fish feeding (Bacheler and Shertzer, 2020; Volkoff and Rønnestad, 2020), growth (Islam et al., 2020; Jin et al., 2021), and physiological metabolism (Sandersfeld et al., 2017; Wen et al., 2017, 2021). In previous studies of the stress response of *C. guichenoti* to temperature, researchers focused on its effect on routine metabolism and its use as a means of physical anesthesia (Luo and Wang, 2012; Zhao, 2014; Zhao et al., 2014). However, with the progress of science and technology, better and more advanced research methods have been developed. For example, transcriptomics and proteomics have been used to study humans (Fagerberg et al., 2014; Cavalli et al., 2020; Chen et al., 2020), animals (Rokyta and Ward, 2017; Panda et al., 2020; Liu Y. et al., 2021), plants (Ramsey et al., 2020; Guo et al., 2021; Xu et al., 2021), and microorganism (Dubrulle et al., 2020; Bassey et al., 2021).

In this study, we used *C. guichenoti* as the experimental object and temperature as the stimulating factor. We applied the combination of transcriptomics and proteomics for the first time to explore the mechanism that underlies high temperature and low temperature tolerance of this fish. Our results highlighted a complex regulatory network of thermal responses, which can be used to further analyze the molecular mechanisms at work in high and low temperature resistance of *C. guichenoti*. Our results also provided some basic data that can be applied to genetic breeding and protect wild *C. guichenoti* after encountering extreme temperature stimulation in the future.

## 3 Methods

### 3.1 Ethics statement

All methods involved in fish collection and treatment were conducted strictly in accordance with the guidelines for animal protection and use (20170226001A) issued by the Fisheries Institute of Sichuan Academy of Agricultural Sciences (Sichuan, China).

### 3.2 Experimental design and sampling

We collected the *C. guichenoti* used in this experiment from the Fisheries Institute, Sichuan Academy of Agricultural Sciences in Sichuan, China. Their body length and weight were 5.71 ± 0.44 g and 1.26 ± 0.27 cm, respectively. Before the start of the formal experiment, the fish were temporarily kept in the laboratory for 7 days, and a pre-experiment was conducted to with 4°C (low temperature, LT), 22°C (normal temperature, NT), and 30°C (high temperature, HT). According to the pre-experimental results, we used LT, NT, and HT for 24 h in the main experiment. Each treatment group contained 30 fish.

The fish were euthanized by a high dose of 3-Aminobenzoic acid ethyl ester methanesulfonate (Sigma-Aldrich, St. Louis, MO, United States) before dissection. We removed brain and gill tissues using sterilized scalpels and scissors. Tissues were quickly placed in liquid nitrogen for quick freezing and then stored in a −80°C freezer until used for analysis.

### 3.3 RNA extraction, library establishment and sequencing

For RNA extraction, 2 g of each tissue sample were thoroughly ground under low temperature, and RNA was extracted according to the instructions of the Invitrogen TRIzol Kit (Thermo Fisher Scientific, Waltham, MA, United States). We detected the concentration and purity of total RNA using a NanoDrop spectrophotometer (Thermo Fisher Scientific), and the integrity was detected using an Agilent 2100 Bioanalyzer and RNA 6000 Nano Kit (Agilent, Santa Clara, CA, United States).

We used 3 μg of RNA samples to construct the libraries. After fragmentation and reverse transcription, 400–500 base pair cDNA was screened for PCR amplification and purification, and finally the RNA library was obtained. After the library was gradually diluted and quantified, we conducted PE150 mode sequencing using the Novaseq 6000 platform (Illumina, San Diego, CA, United States). After quality control, transcriptome assembly, and functional annotation, the sequencing data were used for transcriptome analysis and joint analysis with proteome data (accession number: PRJNA869426; PRJNA869456; PRJNA869436; PRJNA869437).

### 3.4 Protein extraction, library establishment and mass spectrometry

We extracted the protein according to the instructions of the Total Protein Extraction Kit (Nanjing Jiancheng Bioengineering Institute, Jiangsu, China). We tested the quality of 20 μg of protein by SDS-PAGE electrophoresis. After enzymatic hydrolysis of the protein, 100 μg of the product were graded using the reversed-phase high performance liquid chromatography, and all components were collected. Next, 2 μg of peptide segments were removed, and an appropriate amount of iRT standard peptide segments were added for data-dependent acquisition (DDA) and data-independent acquisition (DIA) mass spectrometry detection (Thermo Fisher Scientific). DDA data were directly imported into Spectronaut software (Spectronaut™ 14.4.200727.47784, Biognosys, Switzerland) to build the spectral library. DIA data were also processed using Spectronaut software, and the database was the same as that used for database construction. The generated data were used for proteome analysis and joint analysis with transcriptome data (accession number: PXD036459).

### 3.5 Comparative analysis of differences between transcriptome and proteome

We used the quantitative detection and analysis results of the transcriptome and proteome analyses for the combined transcriptome and proteome analysis. We identified the top 20 differentially expressed genes and proteins, searched for their function in the NCBI database (https://www.ncbi.nlm.nih.gov/), and analyzed and classified the degree of gene-protein association.

### 3.6 Data analysis

Unigenes were annotated in NR (NCBI non-redundant protein sequences, https://www.ncbi.nlm.nih.gov/), GO (Gene Ontology, http://geneontology.org/), KEGG (Kyoto Encyclopedia of Genes and Genomes, http://www.kegg.jp/), Pfam (http://pfam.xfam.org/), eggNOG (Evolutionary Genealogy of Genes: Non-supervised Orthologous Groups, http://eggnog.embl.de/version_3.0/) and Swissprot (http://www.uniprot.org/help/uniprotkb) databases. The data was statistically analyzed by Excel (Microsoft Corporation, Redmond, WA, United States). The principal component analysis (PCA) was performed with SIMCA-P 14.1 (Umetrics, Umea, Sweden). The results are presented as the mean ± standard deviation. Differences were considered to be significant at *p* < 0.05.

## 4 Results

### 4.2 Transcriptome analysis

#### 4.1.1 Transcriptome data statistics

For the high quality sequences in brain tissues, the range at NT was 39,974,412 ± 2,738,421, the range at LT was 39,594,479 ± 856,995, and that at HT was 37,609,588 ± 491,216. In gill tissues, the range at NT was 38,617,540 ± 1,571,668, the range at LT was 41,527,640 ± 3,090,700, and that at HT was 38,393,047 ± 2,819,399. The number of unigene sequences was 201,803, with total length, maximum length, and average length of sequences of 183,256,321, 48,514, and 908.10, respectively. The GC content was 40.72% (Supplementary Table S1).

**FIGURE 1:**
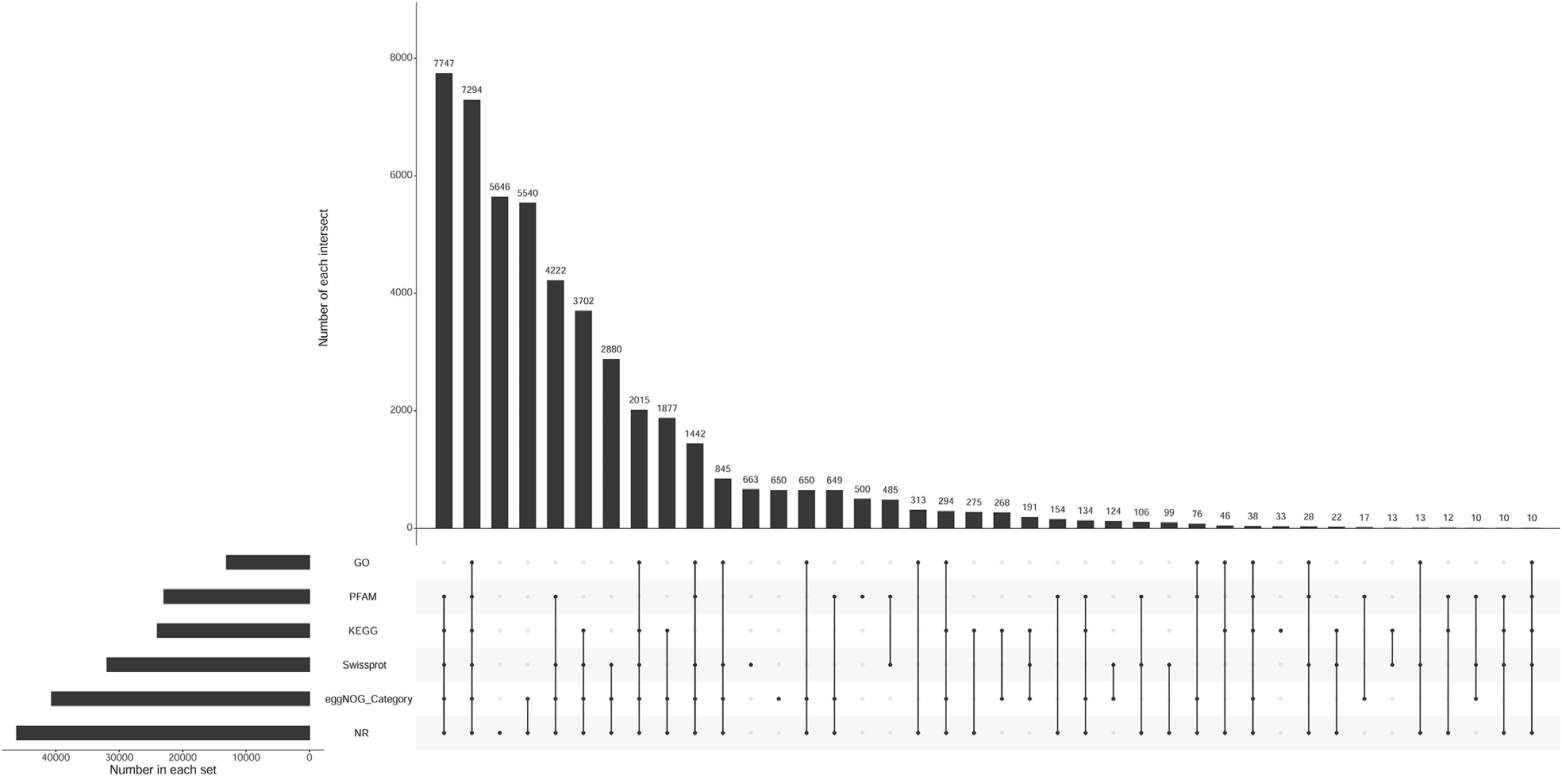
The upset chart of unigenes annotation. Note: Number in each set represents the number of all unigenes annotated to each database. Number of each intersection represents the number of common unigenes annotated by multiple databases. A point on the abscissa represents the number of unique unigenes annotated by each database. The multiple point connection on the abscissa represents the number of common unigenes annotated by multiple databases which were connected.

**FIGURE 2:**
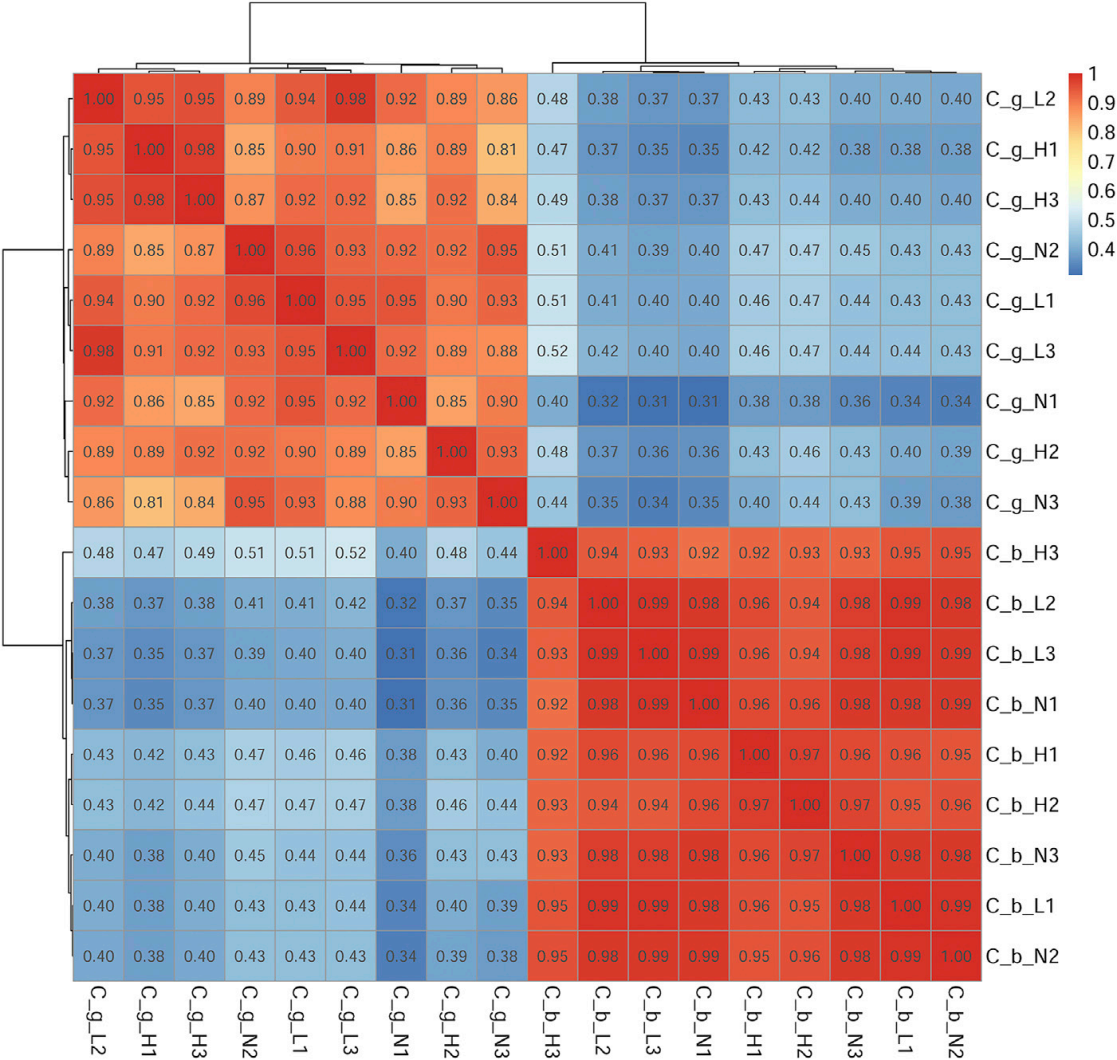
Samples correlation test. Note: C_b_N1, C_b_N2 and C_b_N3 represent brain tissues at 22 °C, C_b_L1, C_b_L2 and C_b_L3 represent brain tissues at 4°C, C_b_H1, C_b_H2 and C_b_H3 represent brain tissues at 30°C; C_g_N1, C_g_N2 and C_g_N3 represent gill tissues at 22°C, C_g_L1, C_g_L2 and C_g_L3 represent gill tissues at 4°C, C_g_H1, C_g_H2 and C_g_H3 represent gill tissues at 30°C. Deeper red represents the higher the correlation, deeper blue represents the lower the correlation. The range of correlation coefficient was 0.8–1, indicating that the correlation among samples were very strong, which below 0.8, indicating that the correlation among samples were low.

The number of unigenes successfully annotated in the NR, GO, KEGG, Pfam, eggNOG, and Swissprot databases were 46,152 (22.87%), 13,077 (6.48%), 23,995 (11.89%), 22,954 (11.37%), 40,671 (20.15%), and 31,934 (15.82%), respectively. In total, 7,294 (3.61%) unigenes were annotated in all databases. Figure 1 shows the annotation data information. The correlation among all gill tissue samples was 0.81–0.98, and the correlation among all brain tissue samples was 0.92–0.99 (Figure 2). The correlation of brain tissue samples was higher than that of gill tissue samples.

#### 4.1.2 Differential expression analysis

Compared with the brain tissues at NT, 408 genes were up- or down-regulated at HT and eight genes were up- or down-regulated at LT. Compared with the gill tissues at NT, 101 genes were up- or down-regulated at HT, and 27 genes were up- or down-regulated at LT (Table 1). The top 20 up- and down-regulated differential expression genes that were described in the NCBI database are summarized in detail in Supplementary Tables S2-S9. In general, the gene expression in brain (average, up 43.31-fold, down 35.84-fold) tissues increased more at HT than at LT relative to NT. In gill tissues at both temperatures, the degree of down-regulation (average, HT 204.67-fold, LT 443.13-fold) was much greater than that of up-regulation (average, HT 28.69-fold, LT 17.68-fold). Only in brain tissues, the number of differential genes increased with HT was 14, and the number of differential genes decreased with HT was 20, the number of differential genes increased with LT was 5, and the number of differential genes decreased with LT was 9. Only in gill tissue, the number of differential genes increased with HT was 14, and the number of differential genes decreased with HT was 7, the number of differential genes increased with LT was 15, and the number of differential genes decreased with LT was 7.

**TABLE 1:** Statistical analysis of differentially expressed genes.

Control_vs._Treat	Up-regulated	Down-regulated	Total
C_b_N_vs._C_b_H	139	269	408
C_b_N_vs._C_b_L	0	8	8
C_b_H_vs._C_b_L	282	256	538
C_g_N_vs._C_g_H	55	46	101
C_g_N_vs._C_g_L	21	6	27
C_g_H_vs._C_g_L	86	73	159

Note: C_b_N represents brain tissues at 22°C, C_b_L represents brain tissues at 4°C, C_b_H represents brain tissues at 30°C; C_g_N represents gill tissues at 22°C, C_g_L represents gill tissues at 4°C, C_g_H represents gill tissues at 30°C. The expression difference of different genes was 10 times, that were, the gene expression in treatment groups were 10 times higher than that of the control groups. *p* < 0.01.

*HSP70* was expressed in all tissues, and the expression level was up-regulated higher at HT than at NT. At both temperatures, the expression in gill tissues was higher than that in brain tissues, but the differential expression in brain tissues (83.11-fold) was higher than that in gill tissues (67.35-fold). Comparing LT with NT revealed that the expression of *HSP70* also increased at LT, but to a lesser extent than that at HT. In brain tissues, the expression of *PLCH2* and *P O 22* increased at HT and decreased at LT. At HT, the expression of *SMC O 3* in brain and gill tissues increased, but the expression in brain tissues was lower than that in gill tissues; at LT, the expression of *SMC O 3* in gill tissues decreased. At HT and LT, *TMPS7* expression increased in brain tissues, but the increase caused by HT was more obvious. At HT, the expression of *MATN1* in brain tissues increased, but the expression decreased at LT. The expression of *APA12* in brain tissues decreased at LT, and that in gill tissues decreased at HT, and the inhibition rate in gill tissues was higher than that of brain tissues.

The expression of *TNR1B* in gill tissues increased at both HT and LT, but the increase caused by HT was greater. The expression of *EGR2B* in gill tissues increased at HT and LT, but the effect at LT was stronger. The expression of *H90A1* in gill tissues increased at both HT and LT. The expression promoted by HT was greater, however, the increase multiple promoted by LT was more. The expression of *IL17F* in gill tissues increased at HT and LT, and the effect of LT was stronger. The expression of *KNG2* in gill tissues decreased significantly at HT and LT. The expression of *FA10A* in gill tissues decreased significantly at HT and LT, and the HT inhibition rate was higher. The expression of *APOA1* in gill tissues decreased significantly at HT and LT, and the inhibition rate at LT was higher. The expression levels of *A1AT*, *FETUA*, *SPYA*, *C1RA*, *FIBG*, *HPPD*, *FIBA*, *PRVB*, and *FIBB* in gill tissues decreased significantly at HT and LT, and the inhibition rates at LT were higher.

#### 4.1.3 KEGG enrichment analysis

According to the results of KEGG enrichment analysis results (Supplementary Figures S1–S4), we selected the top 20 pathways with the most significant enrichment for display. In general, most of the pathways enriched in brain tissues differed among different temperatures; at different temperatures, gill tissues shared more enriched pathways (25%). At HT, brain tissues were mainly enriched in pathways related to metabolism and DNA repair, whereas at LT, they were mainly enriched in cancer-related pathways. At both HT and LT, gill tissues were mainly enriched in pathways related to cell proliferation, apoptosis, immunity, and inflammation.

### 4.2 Proteome analysis

#### 4.2.1 Proteome data

**FIGURE 3:**
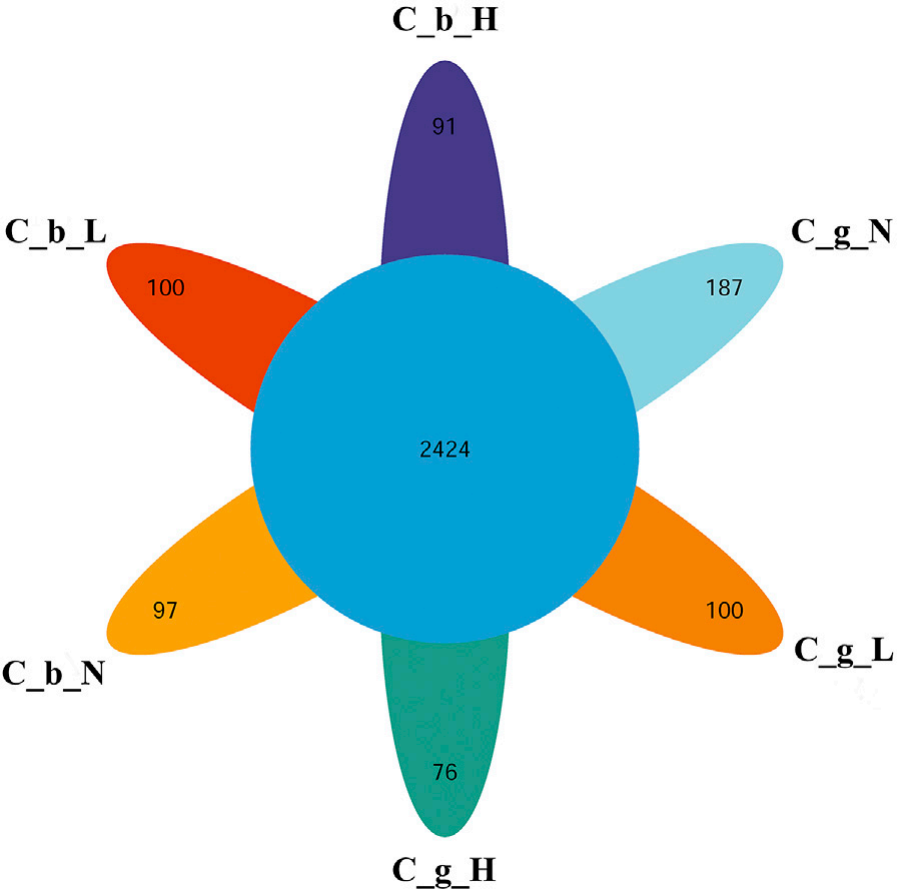
Overlap of DIA data among different tissues and temperature. Note: C_b_N represents brain tissues at 22°C, C_b_L represents brain tissues at 4°C, C_b_H represents brain tissues at 30°C; C_g_N represents gill tissues at 22°C, C_g_L represents gill tissues at 4°C, C_g_H represents gill tissues at 30°C. The number in the center represents the number of common proteins. The number in each petal represents the number of unique proteins.

The identification and quantitative results of the proteome data analysis revealed that the numbers of proteins in brain tissues were 7,431 ± 218 at NT, 7,330 ± 197 at LT, and 5,749 ± 117 at HT. In gill tissues, the range at NT was 6,356 ± 218, the range at LT was 5,072 ± 229, and that at HT was 4,072 ± 442. In total, 10,623 proteins were obtained; 2,424 of them were common to all groups, and the numbers of proteins specific to brain-NT, brain-LT, brain-HT, gill-NT, gill-LT, and gill-HT were 97, 100, 91, 187, 100, and 76 (Figure 3).

**FIGURE 4:**
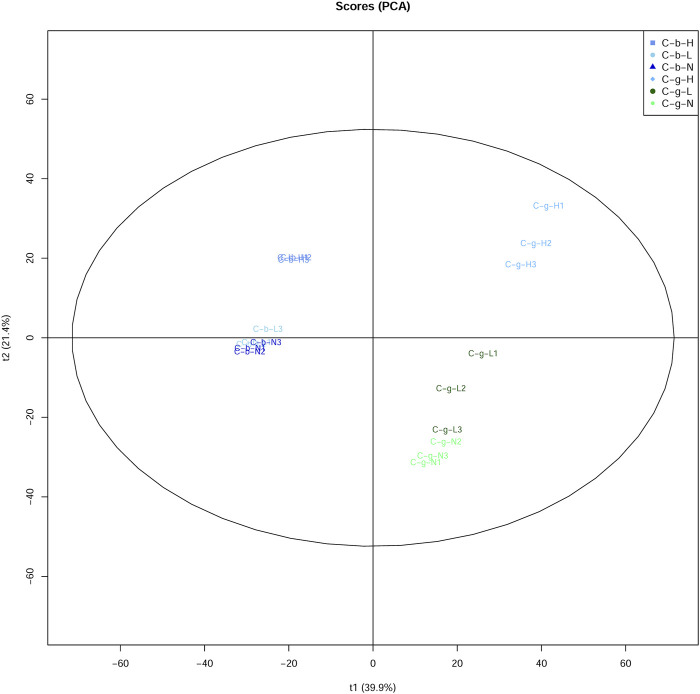
PCA analysis of DIA data among different tissues and temperatures. Note: C_b_N represents brain tissues at 22°C, C_b_L represents brain tissues at 4°C, C_b_H represents brain tissues at 30°C; C_g_N represents gill tissues at 22°C, C_g_L represents gill tissues at 4°C, C_g_H represents gill tissues at 30°C.

The PCA results showed that brain and gill tissues were divided on one side (Figure 4). Comparatively speaking, the aggregation of brain tissues was stronger than that of gill tissues. Additionally, the aggregation of brain and gill tissues was stronger at LT and NT, with the tissues at HT located further away from the tissues under the other temperature conditions.

#### 4.2.2 Differential expression analysis

**FIGURE 5:**
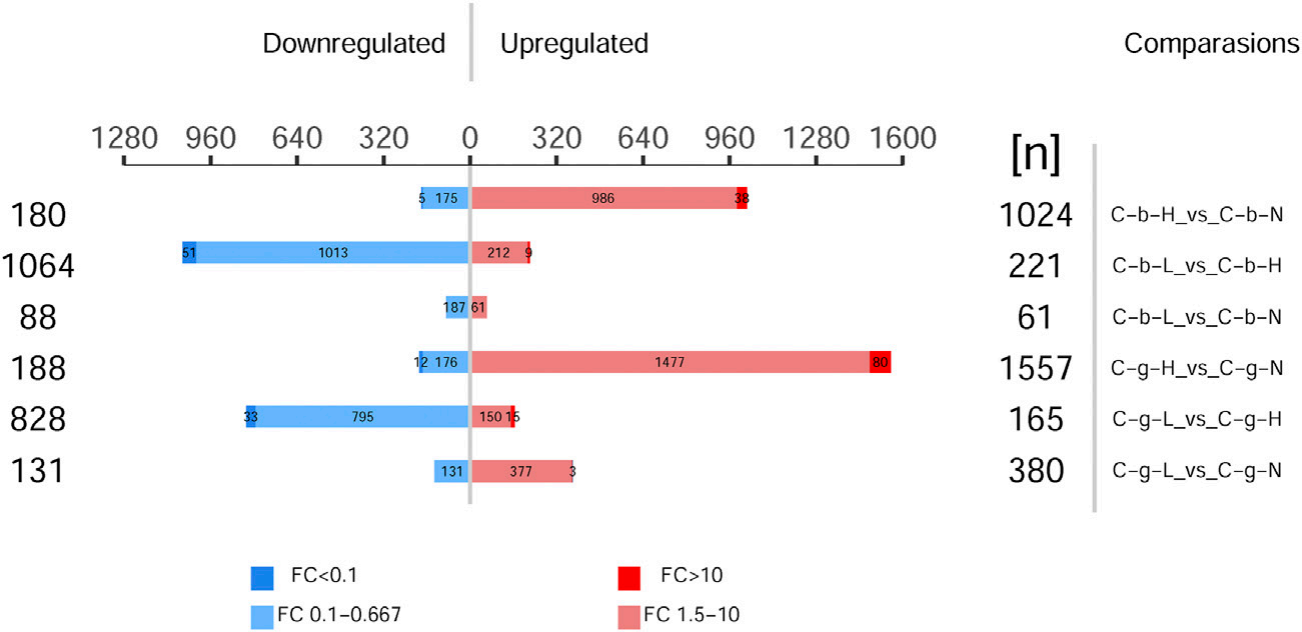
Histogram of differentially expressed proteins among different tissues and temperatures. Note: C_b_N represents brain tissues at 22°C, C_b_L represents brain tissues at 4°C, C_b_H represents brain tissues at 30°C; C_g_N represents gill tissues at 22°C, C_g_L represents gill tissues at 4°C, C_g_H represents gill tissues at 30°C. Comparisons represents difference comparison groups (Treat vs. Control). Upregulated represents Up-regulation of differentially expressed proteins. Downregulated represents Down-regulation of differentially expressed proteins. FC represents fold change. *p* < 0.05.

Figure 5 shows the different protein quantities at different temperatures. The numbers in the treatment vs control comparisons of brain-HT vs brain-NT, brain-LT vs brain-NT, gill-HT vs gill-NT, and gill-LT vs gill-NT were 1,204, 149, 1,745, and 511, respectively. In the brain and gill tissues, the number of up-regulated differentially expressed proteins was greater at HT than at LT. In brain tissues, the number of down-regulated differentially expressed proteins was greater at LT than at HT. In gill tissues, the number of up-regulated differentially expressed proteins was greater at LT as well.

Supplementary Tables S10-17 provide a detailed summary of the top 20 proteins with up- and down-regulation of differentially expressed proteins. In general, the protein expression in brain (average, up 52.67-fold, down 13.54-fold) and gill (average, up 73.02-fold, down 12.92-fold) tissues increased more at HT than at LT. The protein expression in brain (up 3.77-fold, down 4.79-fold) tissues decreased more at LT, and the protein expression in gill (up-8.64-fold, down-4.35-fold) tissues increased more at LT compared to at HT.

At HT, the up-regulated differentially expressed protein with a multiple >100 in brain tissues was ras-related protein Rab-11B (*RB11B*, 229.27-fold), and the down-regulated differentially expressed protein with a multiple >100 was 40S ribosomal protein S9 isoform X2 (*RS9*, 138.63-fold). In gill tissues, the up-regulated differentially expressed proteins with multiples >100 were histone H1.0-like (*AT1A3*, 119.83-fold) and sodium/potassium-transporting ATPase subunit alpha-3-like (*H10B*, 716.84-fold). At LT, only uncharacterized protein LOC557882 (*GSK3B*, 14.78-fold, down-regulated) in brain tissues and 60S ribosomal protein L34-like (*RL34*, 16.70-fold, up-regulated), coronin-1C (*COR1C*, 16.53-fold, up-regulated), and retinoblastoma binding protein 5 (*RBBP5*, 11.41-fold, up-regulated) in gill tissues had multiples >10 and <20; the other proteins had multiples <10.

#### 4.2.3 Subcellular localization analysis

In the subcellular localization analysis (Table 2), the first, second, and fourth types of proteins were Nuclear, Cytoplasmic, and Mitochondrial in all comparison treatment groups. In third place, the Plasma Membrane category and the Extracellular category contained the most proteins for brain tissues and gill tissues, respectively. Proteins in the Cytoskeletal category appeared only in gill tissues.

**TABLE 2:** Statistical analysis of subcellular localization.

Treat vs. control	Nuclear	Cytoplasmic	Plasma membrane	Mitochondrial	Extracellular	Cytoskeletal	Others
C_b_H vs. C_b_N	624	435	162	122	119		30
C_b_L vs. C_b_N	85	46	27	13	21		
C_b_L vs. C_b_H	647	489	180	143	150		28
C_g_H vs. C_g_N	896	700	140	184	210	22	29
C_g_L vs. C_g_N	236	206	46	52	95		14
C_g_L vs. C_g_H	542	378	74	99	114	20	16

Note: C_b_N represents brain tissues at 22°C, C_b_L represents brain tissues at 4°C, C_b_H represents brain tissues at 30°C; C_g_N represents gill tissues at 22°C, C_g_L represents gill tissues at 4°C, C_g_H represents gill tissues at 30°C.

#### 4.2.4 KEGG enrichment analysis

Supplementary Figures S5–S8 show the enrichment maps of the top 20 pathways of differentially expressed proteins. In general, more differentially expressed proteins were enriched in KEEG pathways at HT than at LT. At both HT and LT, more enriched differentially expressed proteins were present in gill tissues than in brain tissues. Many of the differentially expressed proteins were enriched in signaling pathways, such as Endocytosis, PI3K-Akt signaling, and MAPK signaling, in different tissues and at different temperatures. Temperature stimulation strengthened metabolic pathways in brain and gill tissues. At HT relative to NT, pathways related to glucose metabolism were strengthened, and at LT those related to amino acids and fatty acids were strengthened. At both HT and LT, the apoptosis related pathways in brain tissue were strengthened. The analysed results of the differentially expressed protein interaction network are shown in Supplementary Figures S9–S12.

### 4.3 Comparative analysis of differences between transcriptome and proteome

Based on transcriptomics and proteomics data, we extracted and mapped the heat stress-related differentially expressed genes (Table 3). Of the 96 genes we identified as potentially being highly related to temperature stress (59 from transcriptome and 38 from proteome data), *HSP70* was present in both the transcriptome and proteome. In addition, we found that *PLCH2* and *PLCB4* may be involved in the synthesis of 1-phosphatidylinositol 4,5-bisphosphate phosphodiesterase by querying on NCBI, and *RL17* and *RL35* for ribosomal protein, *MYH1*, *MYH13*, and *MYSS* for myosin, *HSP70* and *H90A1* for heat shock protein, *IL20* and *IL17F* for interleukin, and *SYT6* and *SYN3* for synapsin.

**TABLE 3:** The genes that may be highly related to temperature stress.

Gene form transcriptome				Gene form proteome	
Brain		Gill		Brain	Gill
HT	LT	HT	LT	HT	HT
*OST2B*	*PNMA1*	*IL20*	*HA17*	*RB11B*	*H10B*
*HSP70*	*USH2A*	*TNR1B*	*NR3BA*	*RAB3A*	*AT1A3*
*PLCH2*	*BC11B*	*GLMN*	*YCX91*	*DYN3*	*MYH13*
*P O 22*	*FA20A*	*IKKB*	*GVIN1*	*TBCC*	*HMGB1*
*SMC O 3*	*LORF2*	*PEG10*	*ZAN*	*LUM*	*KPYM*
*RL17*	—	*CRYAB*	*GPR54*	*UBAC1*	*HSP70*
*PMP22*	—	*PRLHR*	*SN25B*	*MYH1*	*COR1B*
*5HT3A*	—	*LFG2*	*CASP7*	*PTPRS*	*MYSS*
*SCN2A*	—	*MDH1B*	*EGR3*	*EWS*	*H2AX*
*JUNB*	—	*DNJA4*	*BZW1A*	*EPT1*	*SAR1B*
*SGSM1*	—	*EGR2B*	*IRE1*	*HA10*	*VPP1*
*DESM*	—	*H90A1*	*K1C18*	*NID2*	*RL35*
*UXS3*	—	*NOX O 1*	*RTBS*	*PLCB4*	*PPIL2*
*MY O 15*	—	*TC1A*	*PGAM1*	*SYT6*	*PABPA*
*TMPS7*	—	*IL17F*	*PGH2*	*TB22B*	*TBB1*
*FOXN1*	—	*GT2D2*	*HHLA2*	*CCG1*	*WDR35*
*CASR*	—	*MSS51*	—	*C O 1A1*	*DENR*
*MATN1*	—	*AGO1*	—	*RANG*	*FACE1*
*RBM3*	—	—	—	*AT2A1*	*SYN3*
*MAG*	—	—	—	—	—

Note: HT, represents the tissues at 30°C, LT, represents the tissues at 4°C. Words in italics represent gene names.

At HT, a large number of *HSP70* transcripts and translations were found in brain and gill tissues. This part of the study was based on the results of differential expression of transcriptomics and proteomics and the classical heat stress protein (HSP70 at HT). The results are shown in Supplementary Tables S18, 19. The differential expression multiple of *HSP70* in brain tissues reached 83.11-fold, ranking second, and that of *OST2B* ranked first (155.30-fold); in gill tissues, the differential expression multiple of *HSP70* reached 67.35-fold, ranking third, and those of *IL20* (89.14-fold) and *TNR1B* (71.14-fold) ranked first and second, respectively. The differential expression multiple of HSP70 protein in brain tissues was only 10.84-fold, thus it was not among the top 20, which were *RB11B*, *RAB3A*, *DYN3*, *TBCC*, *LUM*, *UBAC1*, *MYH1*, *PTPRS*, *EWS*, *EPT1*, *HA10*, *NID2*, *PLCB4*, *SYT6*, *TB22B*, *CCG1*, *C O 1A1*, *RANG*, and *AT2A1*, respectively (One protein was not named in NCBI, so only 19 genes were listed). The differential expression multiple of HSP70 protein in gill tissues reached 45.84-fold, ranking sixth; those ranking first through fifth were *H10B*, *ATp1A3*, *MYH13*, *HMGB1*, and *KPYM*, respectively. In this study, we did not evaluate the changes of differentially expressed proteins under LT stimulation in detail because the differential expression multiples was very low (the largest was 16.70-fold).

**FIGURE 6:**
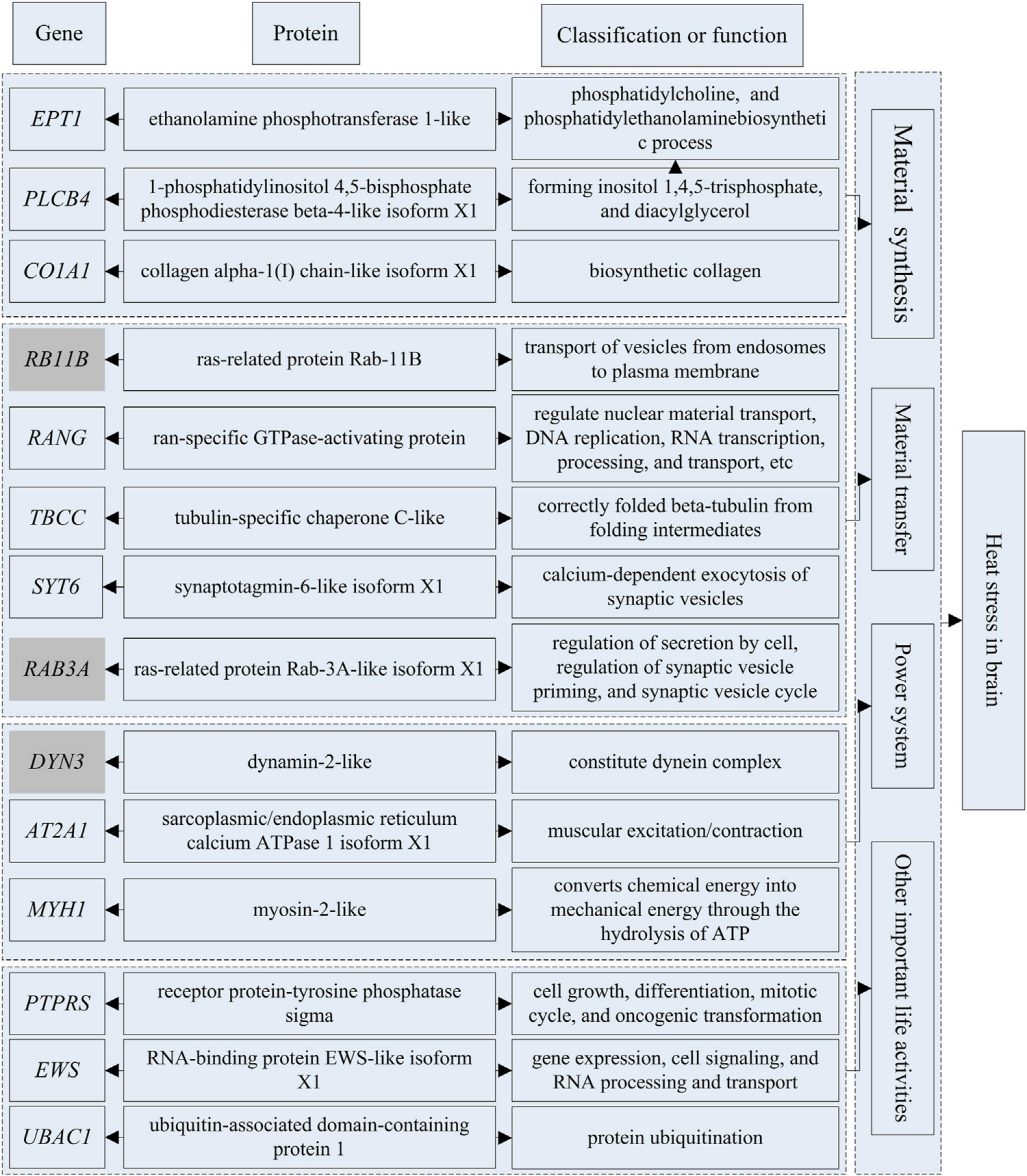
The diagram of gene-protein-potential functions in brain.

**FIGURE 7:**
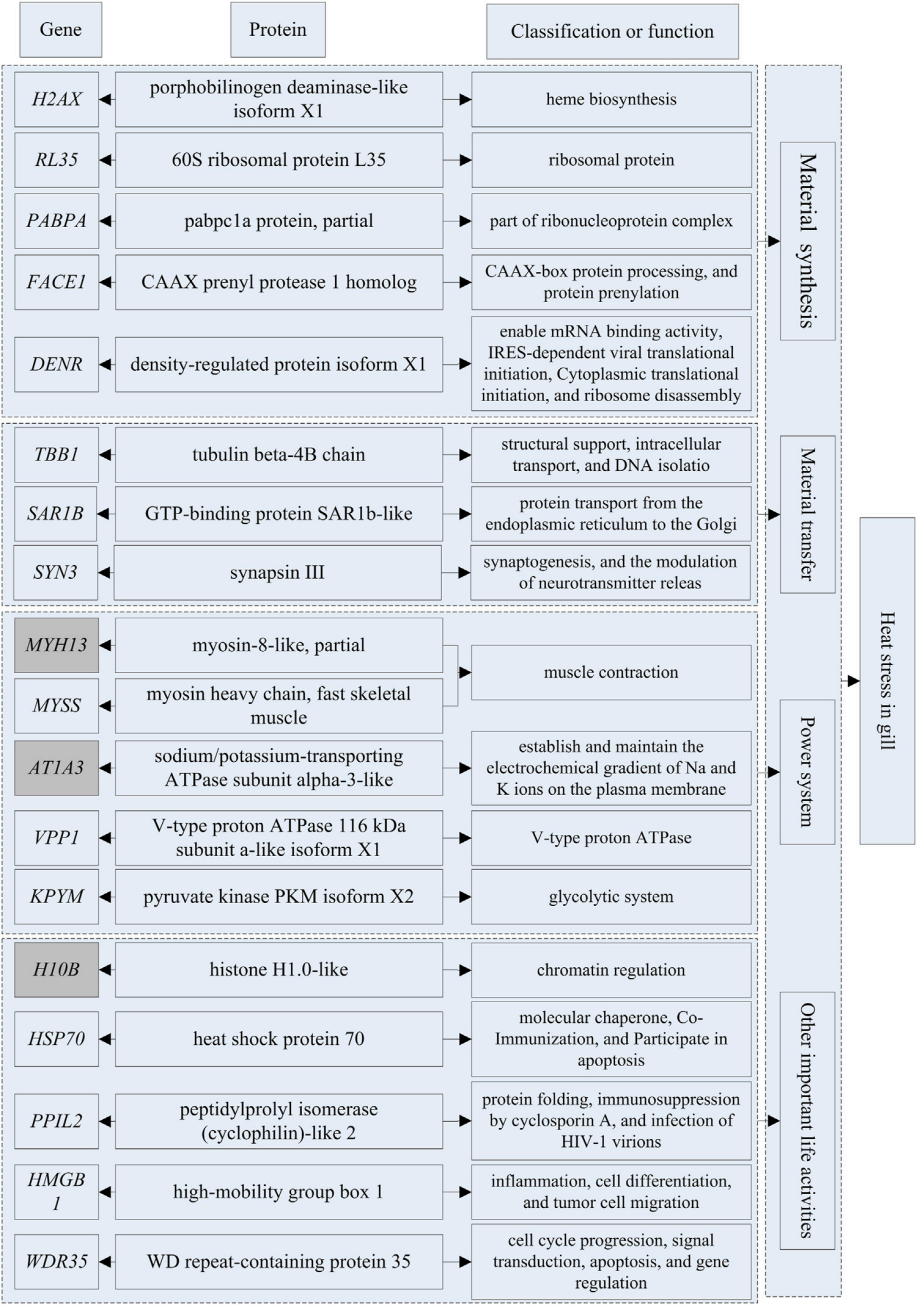
The diagram of gene-protein-potential functions in gill.

The genes that could be searched on NCBI and had specific functional descriptions (Supplementary Tables S18, 19) were extracted and a correlation network diagram was drawn (Figures 6, 7). We classified the genes related to the top 20 proteins into four categories: material synthesis, material transfer, power system, and other important life activities. In brain tissues, two of the top three genes were classified as “material transfer” and one was classified as “power system”. In gill tissues, two of the top three were classified as “power system” and one was classified as “other important life activities” (histone 1-gene regulation, which protects cells and DNA from damage).

## 5 Discussion

Today’s aquatic ecosystem is subject to serious human intervention, which affects important physical and chemical parameters such as water temperature, transparency, and nutrients (Ayele and Atlabachew, 2021). Water temperature variation is an important element with a range of influence on organisms living in it. When facing water temperature changes, fish try to adapt (Papakostas et al., 2014) or leave the area (Valdimarsson et al., 1997), and they may die (Till et al., 2019). The natural habitat of *C. guichenoti* has been negatively impacted by human factors and global warming. Because they cannot escape from their environment, they have to adapt to the abnormal warming phenomenon. Therefore, it is of great significance to study the effects of extreme temperature stimulation of this species.

After fish are stimulated by the outside world, the brain sends instructions to the body so that the tissues can coordinate and cooperate to respond to environmental stimuli. The gills are the respiratory organs of fish, and they are in direct contact with the environment. These tissues are the first to respond to environmental pressure and make adaptive changes. Therefore, we assessed effects of temperature stress on brain and gill tissues in this study.

After being stimulated by temperature, brain and gill tissues of *C. guichenoti* showed certain differences in both transcriptome and proteome data. Overall, the differential expression quantity of both genes and proteins decreased to a certain extent at HT, which was also reported for the transcriptome data from liver tissue of large yellow croaker (*Larimichthys crocea*) (Qian and Xue, 2016). Moreover, the degree of decline in gill tissues was greater than that of in brain tissues in *C. guichenoti*. However, the number of genes decreased more in brain tissues than in gill tissues, while proteins showed the opposite pattern. Comparing the transcriptome and proteome data (Supplementary Tables S2–17) revealed that genes and proteins could be up- or down-regulated in multiples, sometimes hundreds of times, under temperature stimulation. This phenomenon may mean that *C. guichenoti* can resist the negative effects of HT stimulation by reducing the expression of some genes and proteins and increasing the expression of other specific genes and proteins. Hu et al. (2016) found that the number of cold-induced genes in the gills of zebrafish (*Danio rerio*) and tilapia (*Oreochromis niloticus*) increased with the extension of cold treatment time, and similar results were reported for the liver of gilthead sea bream (*Sparus aurata*) (Mininni et al., 2014). Nuez-Ortín et al. (2018) found that HT stimulation caused the differential expression of 276 proteins in the liver of Atlantic salmon (*Salmo salar*), and the differential multiple was 1.2–5.4. These values were smaller than those in our study, which suggests that *C. guichenoti* had a stronger response to the HT stimulation in our study. The correlation analysis of the genome data and the principal component analysis of the proteome data showed that the correlation among different treatment groups was strong for brain tissues and weak for gill tissues. Nitzan et al. (2019) analyzed the transcriptome of gill and liver tissues of blue tilapia (*Oreochromis aureus*) stimulated by LT using the non-metric multidimensional scaling analysis method and found that the similarity of liver tissues was higher than that of gill tissues. Because brain tissues in the fish body are not in contact with the environment, they exist in a more stable environment, whereas the gill tissues are more likely to be affected by external factors, resulting in a weaker correlation.

In this study, the variation laws of differentially expressed genes and proteins were very clear. For genome and proteome and brain and gill tissues, the number of differentially expressed genes or proteins at HT was greater than that at LT, and the number of up-regulated differentially expressed genes or proteins at HT was greater than that at LT. Under stimulation by HT or LT, the number of down-regulated differentially expressed genes in brain tissues was greater than that of up-regulated differentially expressed genes; the number of up-regulated differentially expressed proteins was greater at HT, and the opposite was true at LT. At both HT and LT, the number of up-regulated differentially expressed genes and proteins in gill tissues were greater than that of down-regulated genes and proteins. The number of up-regulated genes induced by LT was greater than that of down-regulated genes, and this phenomenon was also reported for the heart of rainbow trout (*Oncorhynchus mykiss*) and the liver of large yellow croaker (Vornanen et al., 2005; Qian and Xue, 2016). The greater number of up-regulated differentially expressed proteins compared to down-regulated proteins at after HT stimulation was also found in the skin mucus of large yellow croaker (Zhang et al., 2021), but the opposite phenomenon in the liver of rainbow trout (Quan et al., 2021).

In this study, the actual expression abundance of differentially expressed genes was not very high (e.g., *OST2B*, *IL20*, and *TNR1B*), but their up-regulation after temperature stimulation was very significant. This result meant that these genes may play an important role in the temperature stress response in *C. guichenoti*. The actual expression abundance did not affect the function of these genes, which may be due mainly to biological signal amplification *via* the organism-cascade amplification function (Calabrese and Giordano, 2021). We found that the products of the top 20 differentially expressed genes did not all appear in the list of the top 20 differentially expressed proteins, either because these genes were not translated into proteins or because these genes were directly combined with other substances to form new substances when modified after translation. Although some genes are only transcribed to a small extent, the cascade amplification of biological information can result in translation of a lot of protein (i.e., those ranking among the top 20 differentially expressed proteins) to perform biological functions. Indeed, Supplementary Tables S10-17 show that the actual expression abundance of most differentially expressed up-regulated proteins induced by temperature stimulation was very high.

Of the top 20 differentially expressed up-regulated genes identified in our study, only a few have been studied in fish, and most of them were based on other vertebrate organisms. The studies have mainly focused on the nervous system (*PMP22*, *SCN2A*, *5HT3A*, *RBM3*, *IL20*) (Itou et al., 2009; Wang et al., 2010; Bartnik et al., 2011; García-González et al., 2017; Sertel et al., 2021), tumor/cancer (*BC11B*, *JUNB*, *MATN1*, *PEG10*, *PGAM1*, *PRLHR*, *RBM3*, *TNR1B* from the NCBI non-redundant protein sequences) (Heater et al., 2006; Sureban et al., 2008; Abbas et al., 2014; Zhang et al., 2020; Zhu et al., 2020; Liu et al., 2022; Zheng, 2022), the visual system (*MY O 15*, *USH2A*) (Chwalisz et al., 2003; Han et al., 2018), and immune-related aspects (*FOXN1*, *IL20*) (Wang et al., 2010; Lv et al., 2020). In tumors and cancer, the genes are mainly involved in melanoma, acute myeloid leukemia, liver tumor, glioma, breast cancer, colon cancer, bladder cancer, and ovarian cancer, which may suggest that temperature stimulation stress does great harm to organisms. Many studies of immune stress have focused on *HSP70*, including in Japanese sturgeon (*Acipenser schrenckii*) (Luo et al., 2022), Nile tilapia (*O. niloticus*) (Iri et al., 2022), and the ray-finned fish *Schizothorax wangchiachii* (Wang et al., 2022). In our study, the genes stimulated by temperature were mainly concentrated in immune, metabolic, apoptosis, and cancer-related pathways, which was similar to results reported for Atlantic salmon (heat stress) (Nuez-Ortín et al., 2018), pufferfish (*Takifugu fasciatus*) (cold stress) (Wen et al., 2019), and cardinal fish (*Pterapogon kauderni*) (heat and cold stress) (Pan et al., 2021). Our KEEG analysis revealed a large number of genes that have not been specifically studied to date. For example, we found no reports about fish *OST2B*, but its differential expression multiple reached 155.3021-fold, which suggests that it is important for the resistance of *C. guichenoti* to temperature stress. The enrichment pathways of the proteome had similarities with those of the genes, including those related to metabolism, immunity, and apoptosis, but there were differences in categories and quantities as well.

Subcellular localization can indicate the functional regions of proteins (Shi et al., 2012; Lee et al., 2020). In our study, the number of proteins located in each functional part of the cell implied that *C. guichenoti* had a stronger response to HT stimulation than to LT stimulation. The number of proteins in gill tissues was higher than that in brain tissues in most functional parts of the cell, except for the plasma membrane, which may be because the gill tissues were in direct contact with the environmental temperature changes. Specific cytoskeletal structures were the main difference between brain and gill tissues, but other differences were reflected in the plasma membrane and extracellular space. This result suggested that brain tissues may devote more energy to plasma membrane related activities, such as signal transduction, and gill tissues may spend more energy on extracellular secretions, such as mucus. Genes with specific functional descriptions that could be searched in the NCBI database were extracted from the up-regulated differentially expressed proteins, and the top 20 induced by HT stimulation were identified. *Hsp70* is one of the most studied temperature stress proteins, and the differentially expressed proteins located higher than HSP70 in the top 20 list may play important roles in the temperature stress response, but more studies are needed to explore their specific functions.

## 6 Conclusion

When fish are stimulated by temperature, they reduce food intake, show weakened swimming ability, and can even die. In response to temperature stress, a large number of substances change at the molecular level in fish tissues. In other words, the response of fish to temperature stimulation may be attributed to gene transcription and protein expression. When stimulated by temperature, the fish increases or decreases the abundance of some substances at the molecular level, and then adjusts the body state to deal with the adverse environmental impact. Therefore, it is important to explore the differential expression profiles of genes and proteins in *C. guichenoti* after exposure to extreme temperatures.

In this study, the differential expression profiles and relationship network between transcriptome and proteome data revealed that 96 genes, including *OST2B*, *IL20*, *RB11B*, and *H10B*, may play important roles in the response of *C. guichenoti* to temperature stress. The abundance of transcripts or translation products of these genes showed steep changes after being stimulated by temperature, which suggests that they are important in the fish’s resistance to temperature stress. Prior to this study, little was known about the transcriptome and proteome of *C. guichenoti*, so most of the information about the functions of genes and proteins was based on other vertebrates. In this study, we evaluated these genes for the first time in *C. guichenoti*, and we propose that they may play an important role in the protection of germplasm resources, breeding of new varieties, and functional research and verification of special genes and proteins in *C. guichenoti*.

## Data Availability

The original contributions presented in the study are included in the article/Supplementary Material, further inquiries can be directed to the corresponding author. The transcriptome data presented in the study are deposited in the NCBI repository (https://www.ncbi.nlm.nih.gov), accession number are PRJNA869426, PRJNA869456, PRJNA869436 and PRJNA869437. The proteome data presented in the study are deposited in the iProX repository (http://www.iprox.org), accession number is PXD036459.
