# Supplementary material for: Studies on the molecular level changes and potential resistance mechanism of Coreius guichenoti under temperature stimulation
Source: Front Genet. 2022 Oct 3;13:1015505. doi: 10.3389/fgene.2022.1015505 (PMC9574000; doi:10.3389/fgene.2022.1015505)
Supplement: Supplementary file 1 [file Table1.DOCX]

Supplementary Material

# Table

Table S1 Overall statistics of sequences

|  | Transcript | Unigene |
| --- | --- | --- |
| Total length (bp) | 569542043 | 183256321 |
| Sequence number | 439574 | 201803 |
| Max. Length (bp) | 48514 | 48514 |
| Mean Length (bp) | 1295.67 | 908.10 |
| GC% | 42.72 | 40.72 |

Note: Total length (bp) represents the total length of the sequences. Sequence number represents total number of sequences. Max. length (bp) represents maximum length of sequences. Mean length (bp) represents average length of sequences. GC% represents GC content of sequences.

Table S2 The top 20 genes with up-regulation in C_b_H vs C_b_N (Treat vs Control)

| Gene ID | C_b_N | C_b_H | Fold change | Gene length | NR [species name] | Gene name |
| --- | --- | --- | --- | --- | --- | --- |
| TRINITY_DN29885_c0_g1 | 0.3196 | 49.6419 | 155.30 | 1071 | suppressor protein SRP40-like [*Sinocyclocheilus anshuiensis*] | *OST2B* |
| TRINITY_DN7488_c0_g1 | 43.4233 | 3609.1110 | 83.11 | 2546 | heat shock protein 70 [*Coreius guichenoti*] | *HSP70* |
| TRINITY_DN145705_c0_g1 | 0.3062 | 20.9204 | 68.33 | 1177 | 1-phosphatidylinositol 4,5-bisphosphate phosphodiesterase eta-2-like isoform X1 [*Sinocyclocheilus rhinocerous*] | *PLCH2* |
| TRINITY_DN22319_c0_g1 | 10.3252 | 529.2552 | 51.26 | 5482 | uncharacterized protein LOC100192222 [*Danio rerio*] | *PO22* |
| TRINITY_DN2501_c1_g2 | 0.3196 | 15.2384 | 47.67 | 1224 | single-pass membrane and coiled-coil domain-containing protein 3-like [*S. rhinocerous*] | *SMCO3* |
| TRINITY_DN49025_c0_g1 | 0.3196 | 14.8208 | 46.37 | 602 | Rpl17 protein, partial [*Mus musculus*] | *RL17* |
| TRINITY_DN35976_c0_g3 | 0.3196 | 11.5073 | 36.00 | 1686 | peripheral myelin protein 22 isoform 1 [*M. musculus*] | *PMP22* |
| TRINITY_DN142675_c0_g1 | 0.3062 | 10.6241 | 34.70 | 1810 | 5-hydroxytryptamine receptor 3B-like [*Sinocyclocheilus grahami*] | *5HT3A* |
| TRINITY_DN3313_c1_g2 | 0.3523 | 11.6010 | 32.93 | 1124 | LOW QUALITY PROTEIN: sodium channel protein type 1 subunit alpha-like [*S. anshuiensis*] | *SCN2A* |
| TRINITY_DN34757_c0_g1 | 0.3062 | 9.7714 | 31.91 | 1638 | transcription factor jun-B [*M. musculus*] | *JUNB* |
| TRINITY_DN57943_c0_g1 | 0.3196 | 9.9004 | 30.97 | 526 | LOW QUALITY PROTEIN: small G protein signaling modulator 1 [*Callorhinchus milii*] | *SGSM1* |
| TRINITY_DN19805_c0_g3 | 0.6258 | 18.4682 | 29.51 | 2119 | desmin, partial [*M. musculus*] | *DESM* |
| TRINITY_DN156835_c0_g1 | 0.3062 | 8.8109 | 28.78 | 1328 | UDP-glucuronic acid decarboxylase 1 isoform X1 [*Pelodiscus sinensis*] | *UXS3* |
| TRINITY_DN65179_c0_g1 | 0.3196 | 9.0577 | 28.34 | 2022 | mCG6595, isoform CRA_b [*M. musculus*] | *MYO15* |
| TRINITY_DN37114_c0_g1 | 0.6584 | 17.9356 | 27.24 | 1955 | transmembrane protease serine 7 [*D. rerio*] | *TMPS7* |
| TRINITY_DN8492_c0_g1 | 0.3062 | 8.3073 | 27.13 | 3463 | forkhead box protein N1-like [*S. rhinocerous*] | *FOXN1* |
| TRINITY_DN49770_c0_g1 | 2.0483 | 55.4891 | 27.09 | 3826 | extracellular calcium-sensing receptor-like [*S. grahami*] | *CASR* |
| TRINITY_DN13967_c0_g2 | 4.5573 | 123.0691 | 27.00 | 2209 | Matn1 protein, partial [*D. rerio*] | *MATN1* |
| TRINITY_DN88665_c0_g1 | 0.3196 | 8.4146 | 26.32 | 1088 | mCG129611, partial [*M. musculus*] | *RBM3* |
| TRINITY_DN3453_c0_g2 | 0.6258 | 16.4158 | 26.23 | 1617 | sialic acid-binding Ig-like lectin 14 [*S. grahami*] | *MAG* |

Note: C_b_N represents brain tissues at 22 ℃, C_b_H represents brain tissues at 30 ℃. NR represents NCBI non-redundant protein sequences. The expression difference of different genes was 10 times, that were, the gene expression in treatment groups were 10 times higher than that of the control groups. *P*-value was < 0.01.

Table S3 The top 20 genes with down-regulation in C_b_H vs C_b_N (Treat vs Control)

| Gene ID | C_b_N | C_b_H | Fold change | Gene length | NR [species name] | Gene name |
| --- | --- | --- | --- | --- | --- | --- |
| TRINITY_DN6716_c2_g1 | 48.9651 | 0.3437 | 142.45 | 1115 | DNA topoisomerase 2-beta isoform X1 [*D. rerio*] | *TOP2* |
| TRINITY_DN1901_c4_g1 | 59.8637 | 0.6693 | 89.44 | 1628 | SLIT-ROBO Rho GTPase-activating protein 2B-like [*Meleagris gallopavo*] | *SRGP1* |
| TRINITY_DN10906_c2_g1 | 27.0036 | 0.3347 | 80.69 | 1099 | hypothetical protein cypCar_00040571, partial [*Cyprinus carpio*] | *PREX2* |
| TRINITY_DN3345_c6_g1 | 21.1953 | 0.3347 | 63.33 | 388 | sodium/potassium-transporting ATPase subunit alpha-1 [*Manacus vitellinus*] | *AT1A1* |
| TRINITY_DN28344_c1_g1 | 34.4115 | 1.0044 | 34.26 | 2258 | unnamed protein product [*Tetraodon nigroviridis*] | *EPC1* |
| TRINITY_DN8086_c1_g1 | 9.6157 | 0.3170 | 30.33 | 1142 | CUGBP Elav-like family member 4 [*D. rerio*] | *CELF4* |
| TRINITY_DN3994_c11_g1 | 5232.0280 | 193.3241 | 27.06 | 2536 | protein Jumonji-like [*S. rhinocerous*] | *JARD2* |
| TRINITY_DN17245_c0_g2 | 8.2336 | 0.3347 | 24.60 | 531 | tubulin beta-2A chain isoform X6 [*Mandrillus leucophaeus*] | *TBB1* |
| TRINITY_DN22545_c0_g2 | 56.7133 | 2.4061 | 23.57 | 1297 | myelin and lymphocyte protein-like [*S. grahami*] | *MAL* |
| TRINITY_DN147027_c1_g1 | 7.3881 | 0.3347 | 22.08 | 548 | hypothetical protein cypCar_00004021 [*C. carpio*] | *KC2D1* |
| TRINITY_DN4223_c10_g1 | 7.5242 | 0.3437 | 21.89 | 1091 | dual specificity protein phosphatase 26-like isoform X1 [*S. rhinocerous*] | *DUS26* |
| TRINITY_DN132607_c0_g1 | 7.2123 | 0.3437 | 20.98 | 524 | hypothetical protein cypCar_00013446 [*C. carpio*] | *VATA* |
| TRINITY_DN4134_c14_g1 | 6.4731 | 0.3347 | 19.34 | 1157 | EH domain-containing protein 3-like [*S. grahami*] | *EHD3* |
| TRINITY_DN82168_c0_g1 | 145.8436 | 7.6543 | 19.05 | 2747 | - | *FBP1L* |
| TRINITY_DN35465_c2_g1 | 17.7572 | 0.9510 | 18.67 | 1556 | prokineticin-2 precursor [*D. rerio*] | *VAR2* |
| TRINITY_DN5333_c0_g1 | 5.4702 | 0.3170 | 17.26 | 521 | AN1-type zinc finger protein 5-like [*Fundulus heteroclitus*] | *ZFAN5* |
| TRINITY_DN8673_c0_g1 | 27.3084 | 1.6919 | 16.14 | 1142 | - | *LS12A* |
| TRINITY_DN97828_c1_g1 | 15.4651 | 0.9777 | 15.82 | 920 | - | *CDK18* |
| TRINITY_DN155456_c0_g1 | 5.2292 | 0.3437 | 15.21 | 833 | unnamed protein product [*Oncorhynchus mykiss*] | *ZBT8B* |
| TRINITY_DN72797_c0_g1 | 5.0449 | 0.3437 | 14.68 | 1298 | cell death activator CIDE-B [*D. rerio*] | *CIDEB* |

Note: C_b_N represents brain tissues at 22 ℃, C_b_H represents brain tissues at 30 ℃. NR represents NCBI non-redundant protein sequences. The expression difference of different genes was 10 times, that were, the gene expression in treatment groups were 10 times higher than that of the control groups. *P*-value was < 0.01.

Table S4 The top 20 genes with up-regulation in C_b_L vs C_b_N (Treat vs Control)

| Gene ID | C_b_N | C_b_L | Fold change | Gene length | NR [species name] | Gene name |
| --- | --- | --- | --- | --- | --- | --- |
| TRINITY_DN57106_c0_g2 | 0.3527 | 14.4827 | 41.07 | 2091 | uncharacterized protein LOC103911077 [*D. rerio*] | *PNMA1* |
| TRINITY_DN123555_c0_g1 | 0.3080 | 10.0930 | 32.77 | 1783 | usherin-like [*S. anshuiensis*] | *USH2A* |
| TRINITY_DN33393_c0_g4 | 0.3185 | 8.6969 | 27.30 | 1322 | B-cell lymphoma/leukemia 11B isoform X3 [*D. rerio*] | *BC11B* |
| TRINITY_DN3310_c4_g1 | 0.3080 | 5.7064 | 18.53 | 624 | novel protein similar to H.sapiens family with sequence similarity 20 [*D. rerio*] | *FA20A* |
| TRINITY_DN46607_c0_g2 | 0.3185 | 5.6546 | 17.75 | 1160 | LINE-1 reverse transcriptase [*Larimichthys crocea*] | *LORF2* |
| TRINITY_DN37114_c0_g1 | 0.6601 | 10.3011 | 15.59 | 1955 | transmembrane protease serine 7 [*D. rerio*] | *TMPS7* |

Note: C_b_N represents brain tissues at 22 ℃, C_b_L represents brain tissues at 4 ℃. NR represents NCBI non-redundant protein sequences. The expression difference of different genes was 10 times, that were, the gene expression in treatment groups were 10 times higher than that of the control groups. *P*-value was < 0.01.

Table S5 The top 20 genes with down-regulation in C_b_L vs C_b_N (Treat vs Control)

| Gene ID | C_b_N | C_b_L | Fold change | Gene length | NR [species name] | Gene name |
| --- | --- | --- | --- | --- | --- | --- |
| TRINITY_DN2290_c1_g1 | 253.5067 | 11.7077 | 21.65 | 658 | major histocompatibility complex class I-related gene protein-like [*S. anshuiensis*] | *HMR1* |
| TRINITY_DN28472_c0_g1 | 5.3274 | 0.3397 | 15.68 | 524 | tuberin-like protein 1 splicing variant1 [*D. rerio*] | *RGPA1* |
| TRINITY_DN44082_c1_g1 | 5.1147 | 0.3353 | 15.25 | 555 | AFG3-like protein 2 [*Latimeria chalumnae*] | *AFG32* |
| TRINITY_DN8253_c1_g2 | 4.9748 | 0.3397 | 14.65 | 620 | fibroblast growth factor receptor 3-like [*S. grahami*] | *FGFR3* |
| TRINITY_DN31328_c1_g1 | 4.7936 | 0.3353 | 14.30 | 620 | LOW QUALITY PROTEIN: thioredoxin-like protein 4B [*Ornithorhynchus anatinus*] | *TXN4B* |
| TRINITY_DN1252_c1_g1 | 4.7620 | 0.3353 | 14.20 | 1292 | anterior gradient protein 2 homolog precursor [*D. rerio*] | *AGR2* |
| TRINITY_DN18877_c1_g1 | 4.5092 | 0.3397 | 13.27 | 1060 | hypothetical protein cypCar_00022271 [*C. carpio*] | *FAB1A* |
| TRINITY_DN1750_c0_g1 | 49.3336 | 4.4029 | 11.20 | 1404 | apolipoprotein A-I-1 [*Hemibarbus mylodon*] | *APA12* |
| TRINITY_DN5928_c1_g1 | 7.2707 | 0.6706 | 10.84 | 2211 | hypothetical protein cypCar_00002648 [*C. carpio*] | *DMBT1* |
| TRINITY_DN9208_c2_g2 | 10.2057 | 0.9756 | 10.46 | 946 | E3 ubiquitin-protein ligase RING2 [*Anoplopoma fimbria*] | *RING2* |

Note: C_b_N represents brain tissues at 22 ℃, C_b_L represents brain tissues at 4 ℃. NR represents NCBI non-redundant protein sequences. The expression difference of different genes was 10 times, that were, the gene expression in treatment groups were 10 times higher than that of the control groups. *P*-value was < 0.01.

Table S6 The top 20 genes with up-regulation in C_g_H vs C_g_N (Treat vs Control)

| Gene ID | C_g_N | C_g_H | Fold change | Gene length | NR [species name] | Gene name |
| --- | --- | --- | --- | --- | --- | --- |
| TRINITY_DN23732_c0_g1 | 0.3677 | 32.7757 | 89.14 | 792 | interleukin-20-like [*S. grahami*] | *IL20* |
| TRINITY_DN87920_c0_g1 | 3.2380 | 230.3405 | 71.14 | 2076 | tumor necrosis factor receptor superfamily member 1A-like isoform X1 [*S. anshuiensis*] | *TNR1B* |
| TRINITY_DN7488_c0_g1 | 63.2109 | 4257.3370 | 67.35 | 2546 | heat shock protein 70 [*C. guichenoti*] | *HSP70* |
| TRINITY_DN47742_c0_g2 | 0.2964 | 12.3735 | 41.74 | 1603 | glomulin, FKBP associated protein b isoform X1 [*D. rerio*] | *GLMN* |
| TRINITY_DN103095_c0_g1 | 5.8113 | 173.6280 | 29.88 | 1509 | hypothetical protein cypCar_00012958 [*C. carpio*] | *IKKB* |
| TRINITY_DN1156_c2_g2 | 0.2964 | 6.4695 | 21.83 | 443 | Pol polyprotein [*Dicentrarchus labrax*] | *PEG10* |
| TRINITY_DN26445_c5_g1 | 85.8233 | 1847.7680 | 21.53 | 1841 | alpha-crystallin B chain-like [*S. grahami*] | *CRYAB* |
| TRINITY_DN64624_c0_g1 | 1.8618 | 38.8143 | 20.85 | 2766 | prolactin-releasing peptide receptor-like [*S. anshuiensis*] | *PRLHR* |
| TRINITY_DN83532_c0_g1 | 3.7296 | 77.2309 | 20.71 | 2711 | protein lifeguard 2-like [*S. rhinocerous*] | *LFG2* |
| TRINITY_DN23670_c0_g1 | 0.2964 | 6.0543 | 20.43 | 2443 | putative malate dehydrogenase 1B isoform X2 [*D. rerio*] | *MDH1B* |
| TRINITY_DN9714_c0_g2 | 59.5235 | 1144.0900 | 19.22 | 2609 | dnaJ homolog subfamily A member 4-like [*S. anshuiensis*] | *DNJA4* |
| TRINITY_DN5900_c0_g1 | 85.6338 | 1634.8650 | 19.09 | 3108 | hypothetical protein cypCar_00026543 [*C. carpio*] | *EGR2B* |
| TRINITY_DN2501_c0_g1 | 327.5848 | 6243.0450 | 19.06 | 3047 | uncharacterized protein LOC107690531 [*S. anshuiensis*] | *SMCO3* |
| TRINITY_DN861_c0_g1 | 216.3859 | 4114.3580 | 19.01 | 1527 | heat shock protein HSP 90-alpha 1 isoform X1 [*S. grahami*] | *H90A1* |
| TRINITY_DN46809_c0_g1 | 13.8692 | 253.6439 | 18.29 | 2165 | NADPH oxidase organizer 1-like [*S. grahami*] | *NOXO1* |
| TRINITY_DN38630_c0_g1 | 0.3024 | 4.7790 | 15.80 | 710 | transposase domain containing protein [*Lasius niger*] | *TC1A* |
| TRINITY_DN98266_c0_g1 | 5.9178 | 90.3464 | 15.27 | 1124 | interleukin 17C [*Ctenopharyngodon idella*] | *IL17F* |
| TRINITY_DN21647_c1_g1 | 2.4546 | 36.6323 | 14.92 | 2806 | general transcription factor II-I repeat domain-containing protein 2B-like [*Xenopus tropicalis*] | *GT2D2* |
| TRINITY_DN11779_c0_g1 | 23.8371 | 339.2670 | 14.23 | 1956 | putative protein MSS51 homolog, mitochondrial [*S. rhinocerous*] | *MSS51* |
| TRINITY_DN3688_c2_g1 | 0.3677 | 5.2309 | 14.23 | 467 | - | *AGO1* |

Note: C_g_N represents gill tissues at 22 ℃, C_g_H represents gill tissues at 30 ℃. NR represents NCBI non-redundant protein sequences. The expression difference of different genes was 10 times, that were, the gene expression in treatment groups were 10 times higher than that of the control groups. *P*-value was < 0.01.

Table S7 The top 20 genes with down-regulation in C_g_H vs C_g_N (Treat vs Control)

| Gene ID | C_g_N | C_g_H | Fold change | Gene length | NR [species name] | Gene name |
| --- | --- | --- | --- | --- | --- | --- |
| TRINITY_DN41986_c1_g1 | 184.0727 | 0.3029 | 607.79 | 1472 | complement C1q-like protein 2 [*S. rhinocerous*] | *C1QL4* |
| TRINITY_DN176428_c0_g1 | 171.7029 | 0.3451 | 497.61 | 1261 | T-kininogen 2-like [*S. anshuiensis*] | *KNG2* |
| TRINITY_DN11648_c0_g1 | 164.9807 | 0.3505 | 470.71 | 503 | liver-type fatty acid-binding protein [*C. idella*] | *FA10A* |
| TRINITY_DN53554_c0_g1 | 97.4724 | 0.3505 | 278.10 | 794 | hypothetical protein cypCar_00032716, partial [*C. carpio*] | *APOC2* |
| TRINITY_DN7331_c4_g1 | 1925.2670 | 7.2053 | 267.20 | 1102 | apolipoprotein-A-I-2 [*H. mylodon*] | *APOA1* |
| TRINITY_DN1750_c0_g1 | 5063.5080 | 21.1367 | 239.56 | 1404 | apolipoprotein A-I-1 [*H. mylodon*] | *APA12* |
| TRINITY_DN15125_c0_g1 | 131.9097 | 0.6901 | 191.14 | 2003 | carboxylesterase 5A-like [*S. anshuiensis*] | *SASB* |
| TRINITY_DN59591_c1_g1 | 1676.6910 | 9.3817 | 178.72 | 1577 | alpha-1-antitrypsin homolog [*S. grahami*] | *A1AT* |
| TRINITY_DN3116_c0_g1 | 236.3461 | 1.3965 | 169.24 | 1466 | antihemorrhagic factor cHLP-B-like isoform X1 [*S. rhinocerous*] | *FETUA* |
| TRINITY_DN24773_c0_g1 | 260.6340 | 1.7253 | 151.07 | 1641 | serine--pyruvate aminotransferase-like [*S. anshuiensis*] | *SPYA* |
| TRINITY_DN83823_c0_g3 | 50.1819 | 0.3451 | 145.43 | 2484 | complement C1r-A subcomponent-like [*S. rhinocerous*] | *C1RA* |
| TRINITY_DN25288_c0_g1 | 543.8796 | 4.0889 | 133.01 | 1724 | fibrinogen gamma polypeptide [*Hypophthalmichthys molitrix*] | *FIBG* |
| TRINITY_DN161209_c0_g1 | 269.2669 | 2.0444 | 131.71 | 1644 | 4-hydroxyphenylpyruvate dioxygenase [*S. grahami*] | *HPPD* |
| TRINITY_DN39156_c0_g1 | 468.0991 | 3.6431 | 128.49 | 1615 | troponin I, slow skeletal muscle-like [*S. rhinocerous*] | *TNNI3* |
| TRINITY_DN19382_c0_g1 | 326.3060 | 2.9952 | 108.94 | 3493 | inter-alpha-trypsin inhibitor heavy chain H3-like isoform X1 [*S. anshuiensis*] | *ITIH3* |
| TRINITY_DN23431_c0_g1 | 862.9530 | 9.2769 | 93.02 | 2787 | fibrinogen alpha chain-like [*S. anshuiensis*] | *FIBA* |
| TRINITY_DN37630_c0_g1 | 30.3379 | 0.3451 | 87.92 | 1416 | tryptophan 2,3-dioxygenase B isoform X2 [*S. grahami*] | *T23OB* |
| TRINITY_DN157555_c0_g1 | 2313.1520 | 32.0669 | 72.14 | 653 | hypothetical protein cypCar_00020962 [*C. carpio*] | *PRVB* |
| TRINITY_DN1699_c0_g2 | 97.7590 | 1.3802 | 70.83 | 348 | parvalbumin-2 [*S. grahami*] | *PRV2* |
| TRINITY_DN175380_c0_g1 | 689.8049 | 9.7485 | 70.76 | 1948 | fibrinogen beta chain-like [*S. anshuiensis*] | *FIBB* |

Note: C_g_N represents gill tissues at 22 ℃, C_g_H represents gill tissues at 30 ℃. NR represents NCBI non-redundant protein sequences. The expression difference of different genes was 10 times, that were, the gene expression in treatment groups were 10 times higher than that of the control groups. *P*-value was < 0.01.

Table S8 The top 20 genes with up-regulation in C_g_L vs C_g_N (Treat vs Control)

| Gene ID | C_g_N | C_g_L | Fold change | Gene length | NR [species name] | Gene name |
| --- | --- | --- | --- | --- | --- | --- |
| TRINITY_DN54901_c0_g3 | 0.3147 | 11.2089 | 35.62 | 427 | heat shock protein 83-like, partial [*S. rhinocerous*] | *H90A1* |
| TRINITY_DN3664_c0_g2 | 16.8546 | 476.2643 | 28.26 | 402 | MHC class I antigen [*Megalobrama amblycephala*] | *HA17* |
| TRINITY_DN98266_c0_g1 | 6.2601 | 167.3851 | 26.74 | 1124 | interleukin 17C [*C. idella*] | *IL17F* |
| TRINITY_DN5900_c0_g1 | 90.3938 | 2261.6020 | 25.02 | 3108 | hypothetical protein cypCar_00026543 [*C. carpio*] | *EGR2B* |
| TRINITY_DN13037_c0_g1 | 0.3187 | 7.3043 | 22.92 | 475 | LOW QUALITY PROTEIN: neurexin 3 [*Pundamilia nyererei*] | *NR3BA* |
| TRINITY_DN33506_c0_g1 | 1.8712 | 40.3353 | 21.56 | 2145 | hypothetical protein TTRE_0000953901 [*Trichuris trichiura*] | *YCX91* |
| TRINITY_DN343_c1_g1 | 7.1061 | 146.0116 | 20.55 | 1042 | interferon-induced very large GTPase 1-like [*S. grahami*] | *GVIN1* |
| TRINITY_DN37518_c0_g1 | 1.3441 | 26.5260 | 19.74 | 6379 | zonadhesin-like isoform X1 [*D. rerio*] | *ZAN* |
| TRINITY_DN30033_c0_g1 | 0.3187 | 5.4087 | 16.97 | 1849 | G protein-coupled receptor b [*Gobiocypris rarus*] | *GPR54* |
| TRINITY_DN881_c0_g1 | 0.3147 | 5.3035 | 16.85 | 4804 | - | *SN25B* |
| TRINITY_DN2132_c1_g1 | 45.4407 | 732.5445 | 16.12 | 3070 | caspase-3-like [*S. grahami*] | *CASP7* |
| TRINITY_DN227_c2_g1 | 333.4884 | 4474.7060 | 13.42 | 4765 | early growth response protein 3 [*D. rerio*] | *EGR3* |
| TRINITY_DN114383_c0_g1 | 0.7028 | 8.6429 | 12.30 | 938 | basic leucine zipper and w2 domain-containing protein 1-a [*Ictalurus furcatus*] | *BZW1A* |
| TRINITY_DN4923_c0_g1 | 26.3933 | 318.0576 | 12.05 | 2398 | serine/threonine-protein kinase/endoribonuclease ire-1-like isoform X1 [*S. rhinocerous*] | *IRE1* |
| TRINITY_DN87920_c0_g1 | 3.4198 | 40.9452 | 11.97 | 2076 | tumor necrosis factor receptor superfamily member 1A-like isoform X1 [*S. anshuiensis*] | *TNR1B* |
| TRINITY_DN52942_c0_g1 | 6.6969 | 73.6587 | 11.00 | 2685 | LOC553479 protein, partial [*D. rerio*] | *K1C18* |
| TRINITY_DN30738_c1_g1 | 2.4233 | 26.0818 | 10.76 | 1402 | unnamed protein product [*O. mykiss*] | *RTBS* |
| TRINITY_DN6591_c1_g2 | 1.0255 | 10.9849 | 10.71 | 1058 | phosphoglycerate mutase 1 isoform X1 [Ovis aries] | *PGAM1* |
| TRINITY_DN285_c0_g1 | 439.3729 | 4645.6080 | 10.57 | 2525 | prostaglandin G/H synthase 2-like [*S. grahami*] | *PGH2* |
| TRINITY_DN19868_c0_g1 | 48.9633 | 517.6854 | 10.57 | 2080 | programmed cell death 1 ligand 1-like [*S. rhinocerous*] | *HHLA2* |

Note: C_g_N represents gill tissues at 22 ℃, C_g_L represents gill tissues at 4 ℃. NR represents NCBI non-redundant protein sequences. The expression difference of different genes was 10 times, that were, the gene expression in treatment groups were 10 times higher than that of the control groups. *P*-value was < 0.01.

Table S9 The top 20 genes with down-regulation in C_g_L vs C_g_N (Treat vs Control)

| Gene ID | C_g_N | C_g_L | Fold change | Gene length | NR [species name] | Gene name |
| --- | --- | --- | --- | --- | --- | --- |
| TRINITY_DN3488_c0_g2 | 318.4557 | 0.3346 | 951.63 | 4557 | apolipoprotein B-100-like [*S. rhinocerous*] | *APOB* |
| TRINITY_DN25288_c0_g1 | 574.4189 | 0.6136 | 936.21 | 1724 | fibrinogen gamma polypeptide [*H. molitrix*] | *FIBG* |
| TRINITY_DN3664_c0_g3 | 565.6826 | 0.6136 | 921.97 | 522 | MHC class I antigen, partial [*C. carpio*] | *HA1F* |
| TRINITY_DN3116_c0_g1 | 249.5879 | 0.3346 | 745.84 | 1466 | antihemorrhagic factor cHLP-B-like isoform X1 [*S. rhinocerous*] | *FETUA* |
| TRINITY_DN175380_c0_g1 | 728.6008 | 1.0031 | 726.32 | 1948 | fibrinogen beta chain-like [*S. anshuiensis*] | *FIBB* |
| TRINITY_DN40616_c0_g1 | 201.5115 | 0.2797 | 720.45 | 1208 | apolipoprotein A-I-like [*S. rhinocerous*] | *APOEB* |
| TRINITY_DN157555_c0_g1 | 2448.9650 | 4.2365 | 578.06 | 653 | hypothetical protein cypCar_00020962 [*C. carpio*] | *PRVB* |
| TRINITY_DN176428_c0_g1 | 181.3488 | 0.3346 | 541.92 | 1261 | T-kininogen 2-like [*S. anshuiensis*] | *KNG2* |
| TRINITY_DN1750_c0_g1 | 5347.5900 | 10.1003 | 529.45 | 1404 | apolipoprotein A-I-1 [*H. mylodon*] | *APA12* |
| TRINITY_DN59591_c1_g1 | 1770.8450 | 4.1808 | 423.56 | 1577 | alpha-1-antitrypsin homolog [*S. grahami*] | *A1AT* |
| TRINITY_DN7331_c4_g1 | 2032.9350 | 6.6900 | 303.88 | 1102 | apolipoprotein-A-I-2 [*H. mylodon*] | *APOA1* |
| TRINITY_DN11648_c0_g1 | 174.2955 | 0.6693 | 260.42 | 503 | liver-type fatty acid-binding protein [*C. idella*] | *FA10A* |
| TRINITY_DN23431_c0_g1 | 911.4295 | 3.6295 | 251.11 | 2787 | fibrinogen alpha chain-like [*S. anshuiensis*] | *FIBA* |
| TRINITY_DN24773_c0_g1 | 275.3122 | 1.2821 | 214.74 | 1641 | serine--pyruvate aminotransferase-like [*S. anshuiensis*] | *SPYA* |
| TRINITY_DN83823_c0_g3 | 53.02291 | 0.3339 | 158.82 | 2484 | complement C1r-A subcomponent-like [*S. rhinocerous*] | *C1RA* |
| TRINITY_DN161209_c0_g1 | 284.4365 | 2.0071 | 141.72 | 1644 | 4-hydroxyphenylpyruvate dioxygenase [*S. grahami*] | *HPPD* |
| TRINITY_DN1612_c0_g2 | 443.8389 | 3.4056 | 130.33 | 849 | trypsin [*Tribolodon hakonensis*] | *TRY1* |
| TRINITY_DN2745_c2_g1 | 157.5004 | 1.3370 | 117.80 | 1599 | haptoglobin-like isoform X1 [*S. anshuiensis*] | *HPT* |
| TRINITY_DN29591_c0_g1 | 132.7907 | 1.2287 | 108.08 | 3154 | zona pellucida-like domain-containing protein 1 isoform X1 [*S. rhinocerous*] | *ZPLD1* |
| TRINITY_DN14252_c0_g1 | 28.0457 | 0.2797 | 100.27 | 2156 | glycogen [starch] synthase, liver, partial [*S. grahami*] | *GYS2* |

Note: C_g_N represents gill tissues at 22 ℃, C_g_L represents gill tissues at 4 ℃. NR represents NCBI non-redundant protein sequences. The expression difference of different genes was 10 times, that were, the gene expression in treatment groups were 10 times higher than that of the control groups. *P*-value was < 0.01.

Table S10 The top 20 protein with up-regulation in C_b_H vs C_b_N (Treat vs Control)

| Protein ID | Gene name | C_b_H | C_b_N | Fold change | NR [species name] |
| --- | --- | --- | --- | --- | --- |
| TRINITY_DN99_c0_g5.p1 | *RB11B* | 4902.4788 | 21.38 | 229.2738 | ras-related protein Rab-11B [*S. anshuiensis*] |
| TRINITY_DN1495_c2_g1.p1 | *RAB3A* | 6830.0273 | 84.78 | 80.5616 | ras-related protein Rab-3A-like isoform X1 [*S. grahami*] |
| TRINITY_DN4119_c0_g2.p1 | *DYN3* | 1603.0850 | 21.87 | 73.3119 | dynamin-2-like [*S. rhinocerous*] |
| TRINITY_DN18463_c0_g1.p1 | *TBCC* | 1258.3426 | 19.36 | 64.9978 | tubulin-specific chaperone C-like [*S. rhinocerous*] |
| TRINITY_DN175_c0_g2.p1 | *LUM* | 4355.3350 | 72.10 | 60.4072 | hypothetical protein cypCar_00015496 [*C. carpio*]*19* |
| TRINITY_DN681_c2_g1.p1 | *UBAC1* | 1640.0895 | 27.84 | 58.9113 | ubiquitin-associated domain-containing protein 1 [*S. rhinocerous*] |
| TRINITY_DN780_c0_g1.p2 | *MYH1* | 666.1113 | 12.12 | 54.9744 | myosin-2-like [*S. grahami*] |
| TRINITY_DN1510_c0_g1.p1 | *PTPRS* | 943.3145 | 17.61 | 53.5673 | receptor protein-tyrosine phosphatase sigma [*D. rerio*] |
| TRINITY_DN1697_c5_g1.p1 | *EWS* | 681.6288 | 13.84 | 49.2468 | RNA-binding protein EWS-like isoform X1 [*S. anshuiensis*] |
| TRINITY_DN34942_c0_g1.p1 | *EPT1* | 1890.7792 | 40.46 | 46.7338 | ethanolaminephosphotransferase 1-like [*S. rhinocerous*] |
| TRINITY_DN751_c0_g1.p1 | *HA10* | 2882.7831 | 75.30 | 38.2815 | uncharacterized protein LOC107700642 [*S. anshuiensis*] |
| TRINITY_DN6529_c0_g1.p1 | *NID2* | 1714.2909 | 49.26 | 34.7990 | hypothetical protein cypCar_00003088 [*C. carpio*] |
| TRINITY_DN57094_c1_g2.p1 | *PLCB4* | 5153.3721 | 153.41 | 33.5922 | 1-phosphatidylinositol 4,5-bisphosphate phosphodiesterase beta-4-like isoform X1 [*S. anshuiensis*] |
| TRINITY_DN16460_c0_g1.p1 | *TRINITY_DN16460_c0_g1* | 10785.8948 | 338.48 | 31.8653 | protein NLRC3-like [*S. anshuiensis*] |
| TRINITY_DN13208_c0_g1.p1 | *SYT6* | 2936.1214 | 96.62 | 30.3878 | synaptotagmin-6-like isoform X1 [*S. rhinocerous*] |
| TRINITY_DN0_c7_g1.p1 | *TB22B* | 149.5680 | 5.51 | 27.1453 | hypothetical protein cypCar_00024482 [*C. carpio*] |
| TRINITY_DN2275_c2_g1.p1 | *CCG1* | 2326.5427 | 102.49 | 22.7006 | hypothetical protein cypCar_00010553 [*C. carpio*] |
| TRINITY_DN64_c0_g2.p1 | *CO1A1* | 380.2344 | 16.96 | 22.4168 | collagen alpha-1(I) chain-like isoform X1 [*S. anshuiensis*] |
| TRINITY_DN155952_c0_g1.p1 | *RANG* | 6547.8672 | 307.54 | 21.2908 | ran-specific GTPase-activating protein [*Jaculus jaculus*] |
| TRINITY_DN1603_c0_g3.p1 | *AT2A1* | 261.8482 | 13.81 | 18.9576 | sarcoplasmic/endoplasmic reticulum calcium ATPase 1 isoform X1 [*S. anshuiensis*] |

Note: C_b_N represents brain tissues at 22 ℃, C_b_H represents brain tissues at 30 ℃. NR represents NCBI non-redundant protein sequences. *P*-value was < 0.05.

Table S11 The top 20 protein with down-regulation in C_b_H vs C_b_N (Treat vs Control)

| Protein ID | Gene name | C_b_H | C_b_N | Fold change | NR [species name] |
| --- | --- | --- | --- | --- | --- |
| TRINITY_DN43843_c0_g1.p2 | *RS9* | 10.9871 | 1523.1647 | 138.63 | 40S ribosomal protein S9 isoform X2 [*Cricetulus griseus*] |
| TRINITY_DN332_c2_g2.p1 | *CLU* | 6.4830 | 90.1867 | 13.91 | clustered mitochondria protein homolog isoform X1 [*S. grahami*] |
| TRINITY_DN6591_c0_g1.p1 | *PGAM1* | 107.8598 | 1248.0295 | 11.57 | phosphoglycerate mutase 1b [*D. rerio*] |
| TRINITY_DN11770_c0_g1.p1 | *MYO5A* | 158.5749 | 1755.4646 | 11.07 | hypothetical protein cypCar_00026090, partial [*C. carpio*] |
| TRINITY_DN5132_c0_g1.p2 | *GRP78* | 13.1149 | 133.2902 | 10.16 | 78 kDa glucose-regulated protein isoform X3 [*S. anshuiensis*] |
| TRINITY_DN175996_c0_g1.p2 | *ELOB* | 156.6742 | 1284.9310 | 8.20 | transcription elongation factor B polypeptide 2 [*Heterocephalus glaber*] |
| TRINITY_DN367_c1_g1.p1 | *EI2BD* | 13.9739 | 104.4125 | 7.47 | translation initiation factor eIF-2B subunit delta-like [*S. grahami*] |
| TRINITY_DN139135_c0_g1.p1 | *LSM4* | 105.0804 | 744.5111 | 7.09 | U6 snRNA-associated Sm-like protein LSm4 [*Rattus norvegicus*] |
| TRINITY_DN4034_c0_g1.p1 | *OSBL8* | 14.3054 | 98.1323 | 6.86 | oxysterol-binding protein-related protein 8 [*S. rhinocerous*] |
| TRINITY_DN4897_c0_g1.p1 | *GLSK* | 451.2347 | 2979.4052 | 6.60 | glutaminase kidney isoform, mitochondrial-like isoform X1 [*S. anshuiensis*] |
| TRINITY_DN168437_c0_g1.p2 | *RL11* | 44.3702 | 272.9197 | 6.15 | 60S ribosomal protein L11 [*Pteropus alecto*] |
| TRINITY_DN61941_c1_g1.p1 | *SC11A* | 77.1603 | 458.6626 | 5.94 | signal peptidase complex catalytic subunit SEC11A isoform X1 [*S. anshuiensis*] |
| TRINITY_DN8601_c0_g1.p1 | *PA1B2* | 33.5222 | 188.4140 | 5.62 | hypothetical protein cypCar_00023600 [*C. carpio*] |
| TRINITY_DN426_c6_g1.p1 | *SOX1B* | 49.6116 | 247.9963 | 5.00 | transcription factor SOX1B [*D. rerio*] |
| TRINITY_DN35989_c0_g1.p1 | *PXMP4* | 35.8291 | 178.6902 | 4.99 | peroxisomal membrane protein 4 [*S. anshuiensis*] |
| TRINITY_DN588_c1_g1.p1 | *AT1A3* | 551.0498 | 2620.7979 | 4.76 | sodium/potassium-transporting ATPase subunit alpha-3-like [*S. anshuiensis*] |
| TRINITY_DN1915_c3_g1.p1 | *B2MG* | 40.8644 | 178.2943 | 4.36 | beta-2-microglobulin-like [*S. rhinocerous*] |
| TRINITY_DN5928_c5_g1.p1 | *WASC3* | 10.1778 | 43.4094 | 4.27 | WASH complex subunit CCDC53 [*S. rhinocerous*] |
| TRINITY_DN28064_c1_g1.p1 | *EF1A3* | 108.3145 | 443.4543 | 4.09 | elongation factor 1-alpha-like [*S. anshuiensis*] |
| TRINITY_DN3266_c2_g1.p1 | *CAVN2* | 24.6327 | 100.8460 | 4.09 | serum deprivation-response protein [*D. rerio*] |

Note: C_b_N represents brain tissues at 22 ℃, C_b_H represents brain tissues at 30 ℃. NR represents NCBI non-redundant protein sequences. *P*-value was < 0.05.

Table S12 The top 20 protein with up-regulation in C_b_L vs C_b_N (Treat vs Control)

| Protein ID | Gene name | C_b_L | C_b_N | Fold change | NR [species name] |
| --- | --- | --- | --- | --- | --- |
| TRINITY_DN1024_c0_g1.p1 | *MVD1* | 103.5159 | 12.6521 | 8.18 | diphosphomevalonate decarboxylase [*S. rhinocerous*] |
| TRINITY_DN157555_c0_g1.p2 | *PRVB* | 84.6738 | 16.4167 | 5.16 | hypothetical protein cypCar_00020962 [*C. carpio*] |
| TRINITY_DN2531_c0_g2.p1 | *IKKA* | 1055.6770 | 221.3985 | 4.77 | inhibitor of nuclear factor kappa-B kinase subunit alpha [*S. rhinocerous*] |
| TRINITY_DN381_c4_g1.p1 | *HDHD2* | 105.6661 | 22.7219 | 4.65 | haloacid dehalogenase-like hydrolase domain-containing protein 2 [*S. rhinocerous*] |
| TRINITY_DN2745_c2_g1.p1 | *HPT* | 257.1211 | 58.1405 | 4.42 | haptoglobin-like isoform X1 [*S. anshuiensis*] |
| TRINITY_DN38032_c0_g1.p1 | *CNOT9* | 51.3429 | 11.6800 | 4.40 | cell differentiation protein RCD1 homolog isoform X1 [*S. grahami*] |
| TRINITY_DN17471_c0_g1.p1 | *COG5* | 82.6597 | 20.9677 | 3.94 | conserved oligomeric Golgi complex subunit 5-like [*S. anshuiensis*] |
| TRINITY_DN467_c1_g1.p1 | *ACBG2* | 16.2845 | 4.4414 | 3.67 | long-chain-fatty-acid--CoA ligase ACSBG2-like isoform X2 [*S. rhinocerous*] |
| TRINITY_DN81_c1_g2.p1 | *41* | 299.5914 | 84.6887 | 3.54 | protein 4.1 [*D. rerio*] |
| TRINITY_DN7333_c0_g1.p1 | *TRINITY_DN7333_c0_g1* | 73.2284 | 22.3494 | 3.28 | - |
| TRINITY_DN38_c7_g1.p1 | *MUL1* | 71.9842 | 22.6864 | 3.17 | hypothetical protein cypCar_00013648 [*C. carpio*] |
| TRINITY_DN9094_c0_g1.p1 | *SHAN2* | 125.4646 | 40.1044 | 3.13 | SH3 and multiple ankyrin repeat domains protein 2-like [*S. anshuiensis*] |
| TRINITY_DN9397_c0_g1.p1 | *STXA* | 112.4232 | 36.2508 | 3.10 | stonustoxin subunit alpha-like [*S. anshuiensis*] |
| TRINITY_DN790_c2_g1.p1 | *PP4R4* | 53.4218 | 17.5088 | 3.05 | serine/threonine-protein phosphatase 4 regulatory subunit 4-like [*S. rhinocerous*] |
| TRINITY_DN1249_c0_g2.p1 | *TRINITY_DN1249_c0_g2* | 86.4939 | 28.4692 | 3.04 | uncharacterized protein LOC107573998 [*S. grahami*] |
| TRINITY_DN52747_c0_g1.p1 | *MSH2* | 38.8797 | 13.7013 | 2.84 | DNA mismatch repair protein Msh2-like [*S. anshuiensis*] |
| TRINITY_DN4028_c0_g1.p1 | *TBG1* | 257.4014 | 91.3488 | 2.82 | tubulin gamma-2 chain, partial [*Tupaia chinensis*] |
| TRINITY_DN4036_c0_g2.p1 | *CYT* | 81.4605 | 28.9165 | 2.82 | cystatin-like [*S. grahami*] |
| TRINITY_DN10517_c0_g1.p1 | *TRINITY_DN10517_c0_g1* | 39.1725 | 14.0137 | 2.80 | muscle M-line assembly protein unc-89-like [*S. rhinocerous*] |
| TRINITY_DN1648_c1_g1.p1 | *FBCD1* | 27.8093 | 10.1517 | 2.74 | fibrinogen C domain-containing protein 1-like isoform X1 [*S. rhinocerous*] |

Note: C_b_N represents brain tissues at 22 ℃, C_b_L represents brain tissues at 4 ℃. NR represents NCBI non-redundant protein sequences. *P*-value was < 0.05.

Table S13 The top 20 protein with down-regulation in C_b_L vs C_b_N (Treat vs Control)

| Protein ID | Gene name | C_b_L | C_b_N | Fold change | NR [species name] |
| --- | --- | --- | --- | --- | --- |
| TRINITY_DN3486_c0_g1.p1 | *GSK3B* | 19.1156 | 282.4637 | 14.78 | uncharacterized protein LOC557882 [*D. rerio*] |
| TRINITY_DN4131_c3_g1.p1 | *XPO5* | 6.2501 | 41.1143 | 6.58 | exportin-5 [*S. rhinocerous*] |
| TRINITY_DN100497_c0_g3.p1 | *VATB2* | 25.2534 | 160.7408 | 6.37 | ATPase H+ transporting lysosomal V1 subunit B2 [*M. musculus*] |
| TRINITY_DN6569_c0_g2.p1 | *STB1B* | 5.5848 | 34.9418 | 6.26 | histone-lysine N-methyltransferase SETDB1-B-like [*S. grahami*] |
| TRINITY_DN1652_c0_g3.p1 | *MA7D1* | 21.8816 | 123.0566 | 5.62 | MAP7 domain-containing protein 1 isoform X1 [*S. grahami*] |
| TRINITY_DN76_c0_g1.p1 | *RGS3* | 37.0852 | 173.3632 | 4.67 | regulator of G-protein signaling 3-like [*S. grahami*] |
| TRINITY_DN29650_c0_g1.p1 | *ATD1A* | 8.2164 | 37.6941 | 4.59 | ATPase family, AAA domain containing 1a [*D. rerio*] |
| TRINITY_DN22972_c0_g1.p1 | *THTM* | 22.2599 | 101.3901 | 4.55 | 3-mercaptopyruvate sulfurtransferase [*S. grahami*] |
| TRINITY_DN3242_c0_g1.p1 | *TRINITY_DN3242_c0_g1* | 31.3666 | 129.1710 | 4.12 | uncharacterized protein LOC107715042 [*S. rhinocerous*] |
| TRINITY_DN615_c2_g1.p1 | *LNPB* | 39.1507 | 157.9189 | 4.03 | protein lunapark-A-like [*S. rhinocerous*] |
| TRINITY_DN24057_c1_g1.p1 | *MOB2* | 12.3269 | 47.1836 | 3.83 | MOB kinase activator 2 isoform X3 [*S. grahami*] |
| TRINITY_DN15231_c0_g1.p1 | *PHC3* | 7.3949 | 28.2336 | 3.82 | polyhomeotic-like protein 3 [*S. anshuiensis*] |
| TRINITY_DN6695_c0_g4.p1 | *TRINITY_DN6695_c0_g4* | 7.0720 | 26.9875 | 3.82 | putative testis-specific Y-encoded-like protein 3 isoform X1 [*S. grahami*] |
| TRINITY_DN947_c0_g1.p1 | *SGSM1* | 8.2673 | 29.0380 | 3.51 | small G protein signaling modulator 1-like [*S. grahami*] |
| TRINITY_DN642_c0_g4.p1 | *SPTN4* | 37.0690 | 125.9840 | 3.40 | spectrin beta chain, non-erythrocytic 4-like [*S. anshuiensis*] |
| TRINITY_DN416_c3_g1.p1 | *MSI1H* | 32.3919 | 109.2838 | 3.37 | RNA-binding protein Musashi homolog 1-like isoform X1 [*S. anshuiensis*] |
| TRINITY_DN4519_c0_g1.p1 | *ISCU* | 49.1903 | 163.0173 | 3.31 | iron-sulfur cluster assembly enzyme ISCU, mitochondrial-like [*S. grahami*] |
| TRINITY_DN11093_c0_g1.p1 | *RAB24* | 10.2670 | 32.1534 | 3.13 | ras-related protein Rab-24-like [*S. grahami*] |
| TRINITY_DN7470_c0_g1.p1 | *DPYL5* | 306.1448 | 933.5417 | 3.05 | Dpysl5a protein [*D. rerio*] |
| TRINITY_DN3576_c0_g1.p1 | *PRV2* | 51.9376 | 152.5691 | 2.94 | parvalbumin beta-like [*S. anshuiensis*] |

Note: C_b_N represents brain tissues at 22 ℃, C_b_L represents brain tissues at 4 ℃. NR represents NCBI non-redundant protein sequences. *P*-value was < 0.05.

Table S14 The top 20 protein with up-regulation in C_g_H vs C_g_N (Treat vs Control)

| Protein ID | Gene name | C_g_H | C_g_N | Fold change | NR [species name] |
| --- | --- | --- | --- | --- | --- |
| TRINITY_DN1531_c0_g1.p1 | *H10B* | 35814.4648 | 49.9617 | 716.84 | histone H1.0-like [*S. grahami*] |
| TRINITY_DN588_c1_g1.p1 | *AT1A3* | 3842.1855 | 32.0632 | 119.83 | sodium/potassium-transporting ATPase subunit alpha-3-like [*S. anshuiensis*] |
| TRINITY_DN16274_c1_g3.p1 | *MYH13* | 30536.7728 | 314.5783 | 97.07 | myosin-8-like, partial [*Apaloderma vittatum*] |
| TRINITY_DN3703_c5_g1.p1 | *HMGB1* | 7771.5186 | 98.3254 | 79.04 | high-mobility group box 1 [*Carassius auratus* x *M. amblycephala*] |
| TRINITY_DN10118_c0_g1.p1 | *KPYM* | 2990.5980 | 63.4360 | 47.14 | pyruvate kinase PKM isoform X2 [*R. norvegicus*] |
| TRINITY_DN7488_c0_g1.p1 | *HSP70* | 2933.9985 | 64.0058 | 45.84 | heat shock protein 70 [*C. guichenoti*] |
| TRINITY_DN2592_c0_g1.p1 | *TRINITY_DN2592_c0_g1* | 1107.8593 | 28.8859 | 38.35 | hypothetical protein cypCar_00034820 [*C. carpio*] |
| TRINITY_DN506_c2_g1.p1 | *COR1B* | 3174.0454 | 88.3144 | 35.94 | hypothetical protein cypCar_00015898 [*C. carpio*] |
| TRINITY_DN6168_c0_g1.p2 | *MYSS* | 18002.3910 | 602.0210 | 29.90 | myosin heavy chain, fast skeletal muscle [*S. anshuiensis*] |
| TRINITY_DN12475_c0_g1.p1 | *H2AX* | 2941.0567 | 99.4339 | 29.58 | porphobilinogen deaminase-like isoform X1 [*S. rhinocerous*] |
| TRINITY_DN982_c1_g2.p1 | *SAR1B* | 1678.1231 | 60.4983 | 27.74 | GTP-binding protein SAR1b-like [*Astyanax mexicanus*] |
| TRINITY_DN1900_c1_g1.p1 | *VPP1* | 327.6569 | 13.2629 | 24.70 | V-type proton ATPase 116 kDa subunit a-like isoform X1 [*S. grahami*] |
| TRINITY_DN177561_c0_g1.p1 | *RL35* | 3775.2387 | 160.4989 | 23.52 | 60S ribosomal protein L35 [*C. griseus*] |
| TRINITY_DN6114_c1_g1.p1 | *PPIL2* | 2301.5750 | 98.3130 | 23.41 | Peptidylprolyl isomerase (cyclophilin)-like 2 [*D. rerio*] |
| TRINITY_DN1967_c0_g4.p1 | *PABPA* | 12145.0876 | 568.4036 | 21.37 | Pabpc1a protein, partial [*D. rerio*] |
| TRINITY_DN1849_c1_g2.p1 | *TBB1* | 806.0166 | 38.0456 | 21.19 | tubulin beta-4B chain [*A. mexicanus*] |
| TRINITY_DN6402_c0_g1.p1 | *WDR35* | 274.2688 | 13.5165 | 20.29 | WD repeat-containing protein 35 [*S. grahami*] |
| TRINITY_DN10384_c2_g1.p1 | *DENR* | 318.8830 | 15.8097 | 20.17 | density-regulated protein isoform X1 [*Maylandia zebra*] |
| TRINITY_DN2274_c0_g3.p1 | *FACE1* | 845.0885 | 43.9004 | 19.25 | CAAX prenyl protease 1 homolog [*S. grahami*] |
| TRINITY_DN261_c0_g1.p1 | *SYN3* | 171.5290 | 8.9325 | 19.20 | synapsin III [*D. rerio*] |

Note: C_g_N represents gill tissues at 22 ℃, C_g_H represents gill tissues at 30 ℃. NR represents NCBI non-redundant protein sequences. *P*-value was < 0.05.

Table S15 The top 20 protein with down-regulation in C_g_H vs C_g_N (Treat vs Control)

| Protein ID | Gene name | C_g_H | C_g_N | Fold change | NR [species name] |
| --- | --- | --- | --- | --- | --- |
| TRINITY_DN1699_c1_g1.p1 | *PRVB* | 36.4228 | 1150.5286 | 31.59 | parvalbumin [*C. carpio*] |
| TRINITY_DN6324_c1_g1.p1 | *PX11B* | 3.6606 | 87.2123 | 23.82 | peroxisomal membrane protein 11B [*S. anshuiensis*] |
| TRINITY_DN1969_c0_g2.p1 | *SAP* | 11.2032 | 253.2399 | 22.60 | prosaposin isoform X2 [*S. rhinocerous*] |
| TRINITY_DN2590_c0_g1.p1 | *AR8BA* | 20.4772 | 358.2886 | 17.50 | ADP-ribosylation factor-like protein 8B-A [*Poecilia formosa*] |
| TRINITY_DN3092_c1_g1.p1 | *RS12* | 158.0573 | 2759.3665 | 17.46 | 40S ribosomal protein S12 [*P. formosa*] |
| TRINITY_DN121751_c0_g1.p1 | *RS25* | 199.9812 | 2326.6794 | 11.63 | mCG10725, isoform CRA_b, partial [*M. musculus*] |
| TRINITY_DN753_c1_g1.p1 | *UB2V2* | 71.8644 | 825.8060 | 11.49 | ubiquitin-conjugating enzyme E2 variant 2 [*D. rerio*] |
| TRINITY_DN162584_c0_g1.p1 | *FBRL* | 103.7096 | 1183.6409 | 11.41 | FBL protein, partial [*Homo sapiens*] |
| TRINITY_DN1038_c0_g1.p1 | *PROF2* | 72.1922 | 821.6031 | 11.38 | profilin-2-like isoform X1 [*S. grahami*] |
| TRINITY_DN2532_c2_g1.p1 | *FUCL1* | 462.8461 | 5143.4518 | 11.11 | fucolectin-4-like [*S. grahami*] |
| TRINITY_DN1616_c12_g1.p1 | *MKB* | 43.4140 | 452.8802 | 10.43 | midkine-B-like [*S. grahami*] |
| TRINITY_DN453_c3_g1.p1 | *STXB* | 22.8952 | 235.0511 | 10.27 | hypothetical protein cypCar_00048085 [*C. carpio*] |
| TRINITY_DN12487_c0_g1.p1 | *TMOD1* | 16.1855 | 152.6610 | 9.43 | tropomodulin-1 isoform X1 [*S. grahami*] |
| TRINITY_DN3873_c5_g1.p1 | *LDB3* | 39.2351 | 365.3482 | 9.31 | LIM domain-binding protein 3-like [*S. rhinocerous*] |
| TRINITY_DN5393_c0_g1.p1 | *HBB* | 291.5052 | 2700.1329 | 9.26 | hemoglobin beta chain [*Pimephales promelas*] |
| TRINITY_DN2353_c1_g1.p1 | *MYL6* | 155.9750 | 1284.4716 | 8.24 | Zgc:153867 protein [*D. rerio*] |
| TRINITY_DN104620_c0_g1.p1 | *BT3L4* | 57.0707 | 467.5067 | 8.19 | transcription factor BTF3 homolog 4 [*Clupea harengus*] |
| TRINITY_DN9236_c2_g1.p1 | *S100B* | 105.3192 | 841.9101 | 7.99 | hypothetical protein cypCar_00047520 [*C. carpio*] |
| TRINITY_DN23849_c0_g1.p1 | *UCRI* | 76.0350 | 579.9918 | 7.63 | hypothetical protein cypCar_00012311 [*C.* carpio] |
| TRINITY_DN171001_c0_g1.p1 | *ELA1* | 270.0200 | 2047.3841 | 7.58 | pancreatic elastase precursor [*C. idella*] |

Note: C_g_N represents gill tissues at 22 ℃, C_g_H represents gill tissues at 30 ℃. NR represents NCBI non-redundant protein sequences. *P*-value was < 0.05.

Table S16 The top 20 protein with up-regulation in C_b_L vs C_b_N (Treat vs Control)

| Protein ID | Gene name | C_b_L | C_b_N | Fold change | NR [species name] |
| --- | --- | --- | --- | --- | --- |
| TRINITY_DN14548_c0_g1.p1 | *RL34* | 2107.1550 | 126.2099 | 16.70 | 60S ribosomal protein L34-like [*Bubalus bubalis*] |
| TRINITY_DN1056_c0_g2.p1 | *COR1C* | 1855.3273 | 112.2152 | 16.53 | coronin-1C [*D. rerio*] |
| TRINITY_DN2749_c4_g1.p1 | *RBBP5* | 654.2469 | 57.3528 | 11.41 | Retinoblastoma binding protein 5 [*D. rerio*] |
| TRINITY_DN3473_c0_g2.p1 | *RAB6B* | 293.2879 | 29.7921 | 9.84 | hypothetical protein cypCar_00021515, partial [*C. carpio*] |
| TRINITY_DN43410_c0_g5.p1 | *APOB* | 436.7464 | 45.2625 | 9.65 | hypothetical protein cypCar_00029358, partial [*C. carpio*] |
| TRINITY_DN8955_c1_g1.p1 | *FBLN5* | 296.4730 | 30.9470 | 9.58 | fibulin-5-like isoform X1 [*S. anshuiensis*] |
| TRINITY_DN6448_c0_g1.p1 | *ACTN2* | 210.1753 | 23.4638 | 8.96 | alpha-actinin-2-like [*S. anshuiensis*] |
| TRINITY_DN3844_c0_g1.p1 | *LIN7C* | 251.8360 | 30.3124 | 8.31 | protein lin-7 homolog C [*S. grahami*] |
| TRINITY_DN1046_c0_g1.p1 | *TB10C* | 2034.8835 | 254.5013 | 8.00 | uncharacterized protein LOC107557678 [*S. grahami*] |
| TRINITY_DN64646_c0_g1.p1 | *SC61B* | 1761.7472 | 226.6918 | 7.77 | mCG114989, partial [*M. musculus*] |
| TRINITY_DN21225_c0_g1.p1 | *TRINITY_DN21225_c0_g1* | 3952.3533 | 526.1283 | 7.51 | hypothetical protein cypCar_00003012 [*C. carpio*] |
| TRINITY_DN897_c0_g2.p1 | *IF5* | 526.9117 | 71.9232 | 7.33 | eukaryotic translation initiation factor 5-like [*S. anshuiensis*] |
| TRINITY_DN1725_c1_g1.p1 | *VINC* | 266.1577 | 39.7016 | 6.70 | vinculin isoform X2 [*S. anshuiensis*] |
| TRINITY_DN12729_c0_g1.p1 | *BABA1* | 126.2308 | 19.1596 | 6.59 | Zgc:100909 [*D. rerio*] |
| TRINITY_DN132_c0_g4.p1 | *CANX* | 495.2624 | 76.2314 | 6.50 | calpain-1 catalytic subunit-like [*S. anshuiensis*] |
| TRINITY_DN1387_c1_g1.p1 | *143BA* | 2796.1346 | 433.6982 | 6.45 | 14-3-3 protein beta/alpha-A-like [*S. grahami*] |
| TRINITY_DN4228_c0_g1.p1 | *COSA1* | 337.9861 | 53.8280 | 6.28 | collagen alpha-1(XXVIII) chain-like, partial [*S. rhinocerous*] |
| TRINITY_DN306_c3_g1.p1 | *K154L* | 5103.5087 | 816.7009 | 6.25 | UPF0606 protein KIAA1549L-like isoform X2 [*S. grahami*] |
| TRINITY_DN95871_c0_g1.p1 | *EIF3E* | 469.4355 | 75.1983 | 6.24 | eukaryotic translation initiation factor 3 subunit E isoform X1 [*Papio anubis*] |
| TRINITY_DN346_c1_g1.p1 | *SMCE1* | 141.3962 | 22.6959 | 6.23 | SWI/SNF-related matrix-associated actin-dependent regulator of chromatin subfamily E member 1 [*D. rerio*] |

Note: C_g_N represents gill tissues at 22 ℃, C_g_L represents gill tissues at 4 ℃. NR represents NCBI non-redundant protein sequences. *P*-value was < 0.05.

Table S17 The top 20 protein with down-regulation in C_b_L vs C_b_N (Treat vs Control)

| Protein ID | Gene name | C_b_L | C_b_N | Fold change | NR [species name] |
| --- | --- | --- | --- | --- | --- |
| TRINITY_DN2379_c0_g1.p1 | *LADD* | 3333.1943 | 29078.7350 | 8.72 | hypothetical protein cypCar_00026293 [*C. carpio*] |
| TRINITY_DN114473_c0_g4.p1 | *RL3* | 132.3163 | 806.4976 | 6.10 | 60S ribosomal protein L3 [*M. musculus*] |
| TRINITY_DN6353_c0_g1.p1 | *IF4EA* | 60.7878 | 334.2688 | 5.50 | hypothetical protein cypCar_00041557 [*C. carpio*] |
| TRINITY_DN1223_c0_g1.p1 | *HERC4* | 27.7638 | 137.1924 | 4.94 | probable E3 ubiquitin-protein ligase HERC4 [*S. rhinocerous*] |
| TRINITY_DN35989_c0_g1.p1 | *PXMP4* | 35.6808 | 163.6051 | 4.59 | peroxisomal membrane protein 4 [*S. anshuiensis*] |
| TRINITY_DN42353_c0_g1.p1 | *SAM9L* | 16.7575 | 73.7878 | 4.40 | sterile alpha motif domain-containing protein 9-like [*S. rhinocerous*] |
| TRINITY_DN20355_c0_g1.p1 | *EPD* | 77.9143 | 336.0809 | 4.31 | ependymin [*S. grahami*] |
| TRINITY_DN7006_c0_g1.p1 | *TRI29* | 14.7100 | 62.4636 | 4.25 | tripartite motif-containing protein 16-like, partial [*S. rhinocerous*] |
| TRINITY_DN4953_c0_g1.p1 | *KDM4A* | 34.5675 | 144.9105 | 4.19 | lysine-specific demethylase 4A-like isoform X1 [*S. anshuiensis*] |
| TRINITY_DN3185_c0_g1.p1 | *STXB* | 90.1994 | 370.1174 | 4.10 | NACHT, LRR and PYD domains-containing protein 1-like [*S. rhinocerous*] |
| TRINITY_DN18566_c0_g1.p1 | *RHBL2* | 17.5835 | 71.9836 | 4.09 | rhomboid-related protein 2 isoform X1 [*D. rerio*] |
| TRINITY_DN972_c0_g1.p1 | *GPCP1* | 19.3658 | 77.2348 | 3.99 | glycerophosphocholine phosphodiesterase GPCPD1-like [*S. anshuiensis*] |
| TRINITY_DN6093_c0_g1.p1 | *ZO2* | 44.7494 | 176.0377 | 3.93 | tight junction protein ZO-2-like isoform X5 [*S. grahami*] |
| TRINITY_DN9925_c0_g2.p1 | *RL32* | 250.7666 | 905.7013 | 3.61 | 60S ribosomal protein L32-like [*S. anshuiensis*] |
| TRINITY_DN2332_c2_g1.p1 | *RS19* | 530.0225 | 1888.9853 | 3.56 | 40S ribosomal protein S19-like [*S. anshuiensis*] |
| TRINITY_DN25154_c0_g1.p1 | *DDX42* | 37.0347 | 128.7429 | 3.48 | ATP-dependent RNA helicase DDX42-like [*S. anshuiensis*] |
| TRINITY_DN12267_c0_g1.p1 | *TRINITY_DN12267_c0_g1* | 66.8748 | 227.4462 | 3.40 | proteoglycan 4-like [*S. grahami*] |
| TRINITY_DN6610_c0_g1.p1 | *GIMA8* | 16.4832 | 54.9002 | 3.33 | GTPase IMAP family member 7-like [*S. anshuiensis*] |
| TRINITY_DN24758_c0_g3.p1 | *TRINITY_DN24758_c0_g3* | 40.2800 | 133.2800 | 3.31 | hemicentin-2-like [*S. grahami*] |
| TRINITY_DN145033_c0_g2.p1 | *METK2* | 218.2004 | 713.2072 | 3.27 | S-adenosylmethionine synthase isoform type-2-like [*L. crocea*] |

Note: C_g_N represents gill tissues at 22 ℃, C_g_L represents gill tissues at 4 ℃. NR represents NCBI non-redundant protein sequences. *P*-value was < 0.05.

Table S18 Association analysis between transcriptome and proteome based on *HSP70* with heat stress in brain

| Transcriptomics | | proteomics | | |
| --- | --- | --- | --- | --- |
| Gene name | Fold change  (C_b_H/C_b_N) | Gene Name | Fold change  (C-b-H/C-b-N) | NR [species name] |
| *OST2B* | 155.30 | *RB11B* | 229.27 | ras-related protein Rab-11B |
| *HSP70* | 83.11 | *RAB3A* | 80.56 | ras-related protein Rab-3A-like isoform X1 |
|  |  | *DYN3* | 73.31 | dynamin-2-like |
|  |  | *TBCC* | 65.00 | tubulin-specific chaperone C-like |
|  |  | *LUM* | 60.41 | hypothetical protein cypCar_00015496 |
|  |  | *UBAC1* | 58.91 | ubiquitin-associated domain-containing protein 1 |
|  |  | *MYH1* | 54.97 | myosin-2-like |
|  |  | *PTPRS* | 53.57 | receptor protein-tyrosine phosphatase sigma |
|  |  | *EWS* | 49.25 | RNA-binding protein EWS-like isoform X1 |
|  |  | *EPT1* | 46.73 | ethanolamine phosphotransferase 1-like |
|  |  | *HA10* | 38.28 | uncharacterized protein LOC107700642 |
|  |  | *NID2* | 34.80 | hypothetical protein cypCar_00003088 |
|  |  | *PLCB4* | 33.59 | 1-phosphatidylinositol 4,5-bisphosphate phosphodiesterase beta-4-like isoform X1 |
|  |  | *TRINITY_DN16460_c0_g1* | 31.87 | protein NLRC3-like |
|  |  | *SYT6* | 30.39 | synaptotagmin-6-like isoform X1 |
|  |  | *TB22B* | 27.15 | hypothetical protein cypCar_00024482 |
|  |  | *CCG1* | 22.70 | hypothetical protein cypCar_00010553 |
|  |  | *CO1A1* | 22.42 | collagen alpha-1(I) chain-like isoform X1 |
|  |  | *RANG* | 21.29 | ran-specific GTPase-activating protein |
|  |  | *AT2A1* | 18.96 | sarcoplasmic/endoplasmic reticulum calcium ATPase 1 isoform X1 |
|  |  | *HSP70* | 10.84 | heat shock protein 70 |

Note: Data were obtained from differential expression analysis of transcriptome and proteome..

Table S19 Association analysis between transcriptome and proteome based on *HSP70* with heat stress in gill

| transcriptomics | | proteomics | | |
| --- | --- | --- | --- | --- |
| Gene name | Fold change (C_g_H/C_g_N) | Gene Name | Fold change (C-g-H/C-g-N) | NR [species name] |
| *IL20* | 89.12 | *H10B* | 716.84 | histone H1.0-like |
| *TNR1B* | 71.14 | *AT1A3* | 119.83 | sodium/potassium-transporting ATPase subunit alpha-3-like |
| *HSP70* | 67.35 | *MYH13* | 97.07 | myosin-8-like, partial |
|  |  | *HMGB1* | 79.04 | high-mobility group box 1 |
|  |  | *KPYM* | 47.14 | pyruvate kinase PKM isoform X2 |
|  |  | *HSP70* | 45.84 | heat shock protein 70 |
|  |  | *TRINITY_DN2592_c0_g1* | 38.35 | hypothetical protein cypCar_00034820 |
|  |  | *COR1B* | 35.94 | hypothetical protein cypCar_00015898 |
|  |  | *MYSS* | 29.90 | myosin heavy chain, fast skeletal muscle |
|  |  | *H2AX* | 29.58 | porphobilinogen deaminase-like isoform X1 |
|  |  | *SAR1B* | 27.74 | GTP-binding protein SAR1b-like |
|  |  | *VPP1* | 24.70 | V-type proton ATPase 116 kDa subunit a-like isoform X1 |
|  |  | *RL35* | 23.52 | 60S ribosomal protein L35 |
|  |  | *PPIL2* | 23.41 | Peptidylprolyl isomerase (cyclophilin)-like 2 |
|  |  | *PABPA* | 21.37 | Pabpc1a protein, partial |
|  |  | *TBB1* | 21.19 | tubulin beta-4B chain |
|  |  | *WDR35* | 20.29 | WD repeat-containing protein 35 |
|  |  | *DENR* | 20.17 | density-regulated protein isoform X1 |
|  |  | *FACE1* | 19.25 | CAAX prenyl protease 1 homolog |
|  |  | *SYN3* | 19.20 | synapsin III |

Note: Data were obtained from differential expression analysis of transcriptome and proteome.

# Figures


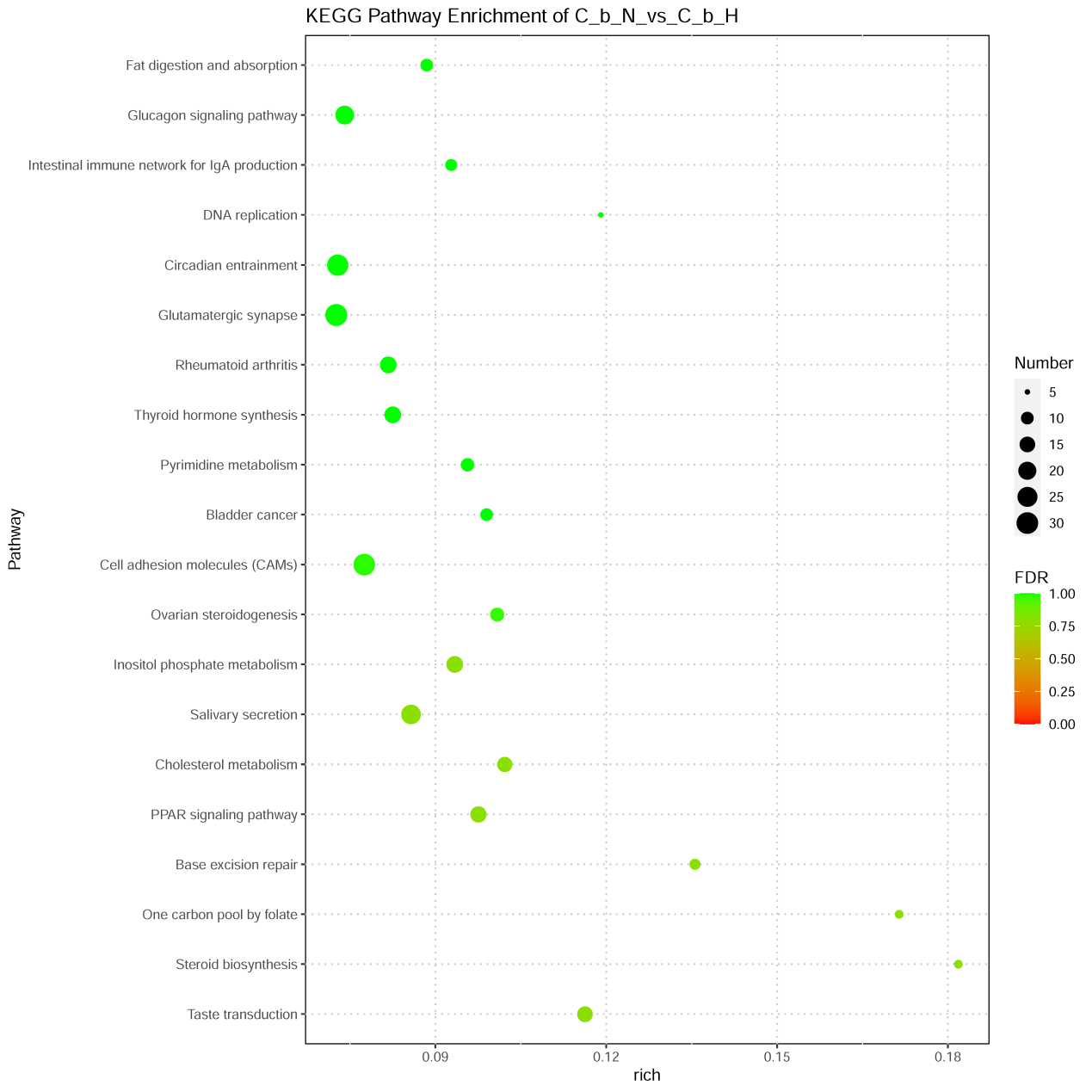


Fig. S1 The top 20 KEEG pathway enrichment of C_b_H vs C_b_N (Treat vs Control)

Note: C_b_N represents brain tissues at 22 ℃, C_b_H represents brain tissues at 30 ℃. Rich factor: [the number of differential genes enriched in the pathway]/[the number of differential genes annotated], the enrichment degree increases with the increase of rich. The general value range of FDR is 0 - 1, the enrichment degree increases with the decrease of FDR. Number represents the number of genes enriched in the pathway.


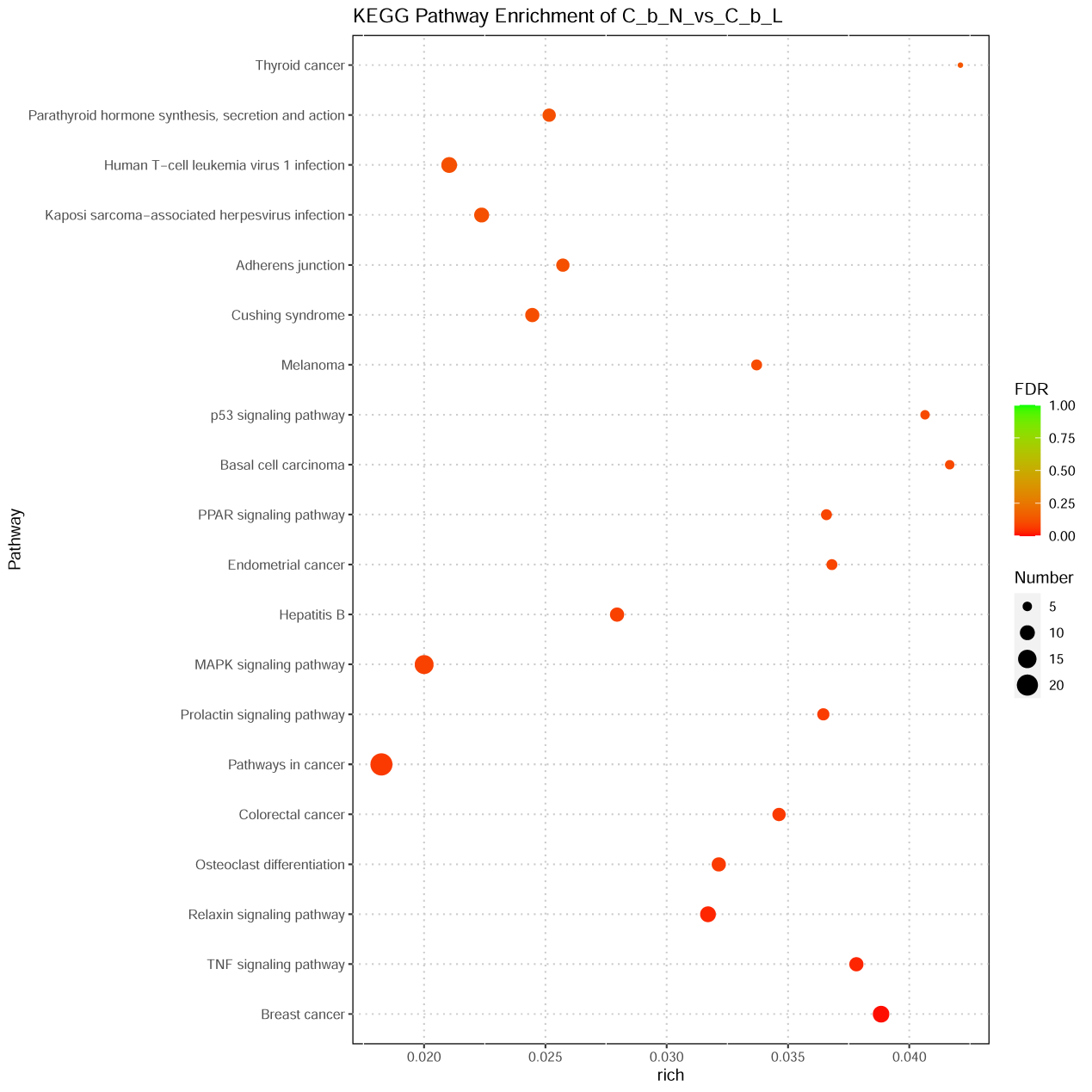


Fig. S2 The top 20 KEEG pathway enrichment of C_b_L vs C_b_N (Treat vs Control)

Note: C_b_N represents brain tissues at 22 ℃, C_b_L represents brain tissues at 4 ℃. Rich factor: [the number of differential genes enriched in the pathway]/[the number of differential genes annotated], the enrichment degree increases with the increase of rich. The general value range of FDR is 0 - 1, the enrichment degree increases with the decrease of FDR. Number represents the number of genes enriched in the pathway.


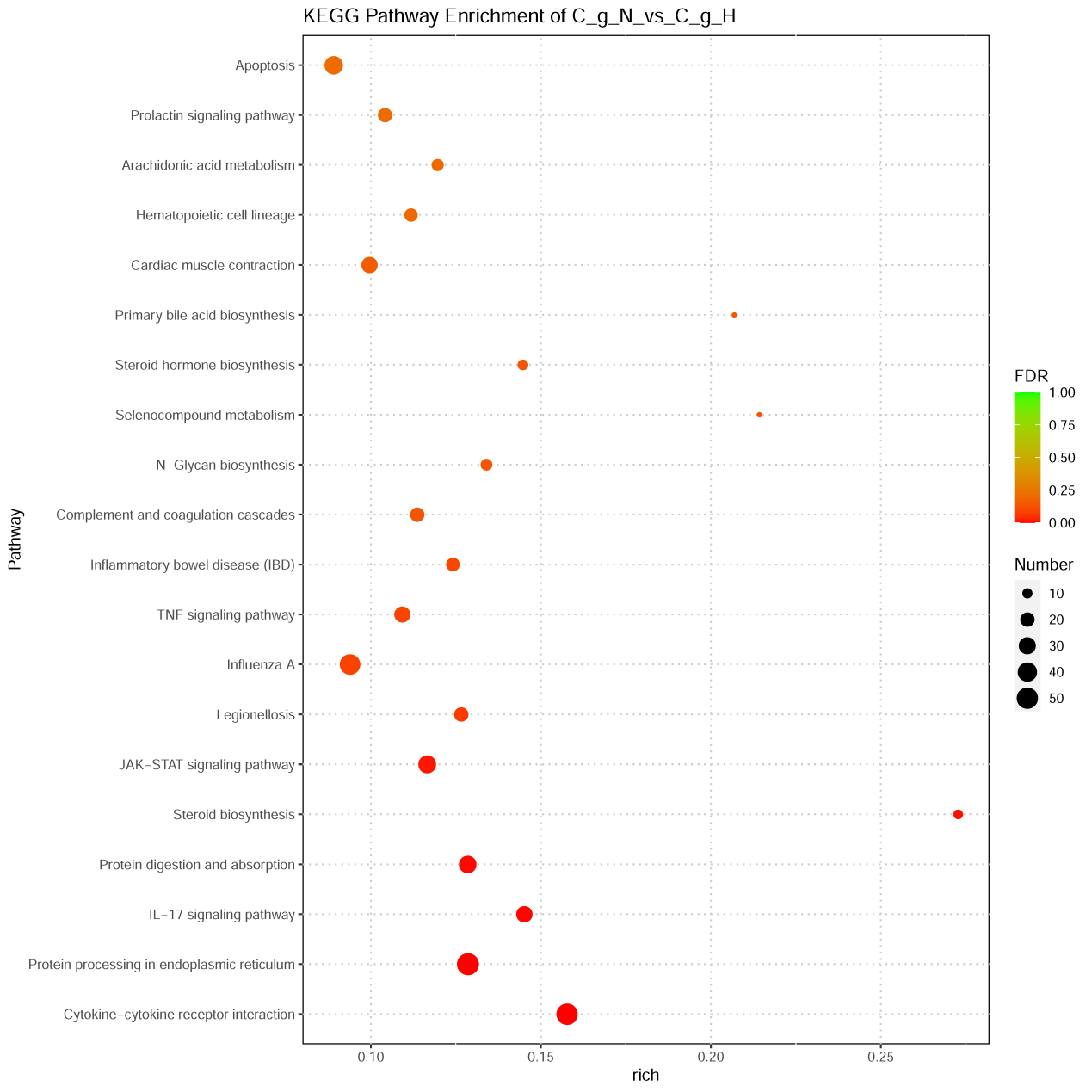


Fig. S3 The top 20 KEEG pathway enrichment of C_g_H vs C_g_N (Treat vs Control)

Note: C_g_N represents brain tissues at 22 ℃, C_g_H represents brain tissues at 30 ℃. Rich factor: [the number of differential genes enriched in the pathway]/[the number of differential genes annotated], the enrichment degree increases with the increase of rich. The general value range of FDR is 0 - 1, the enrichment degree increases with the decrease of FDR. Number represents the number of genes enriched in the pathway.


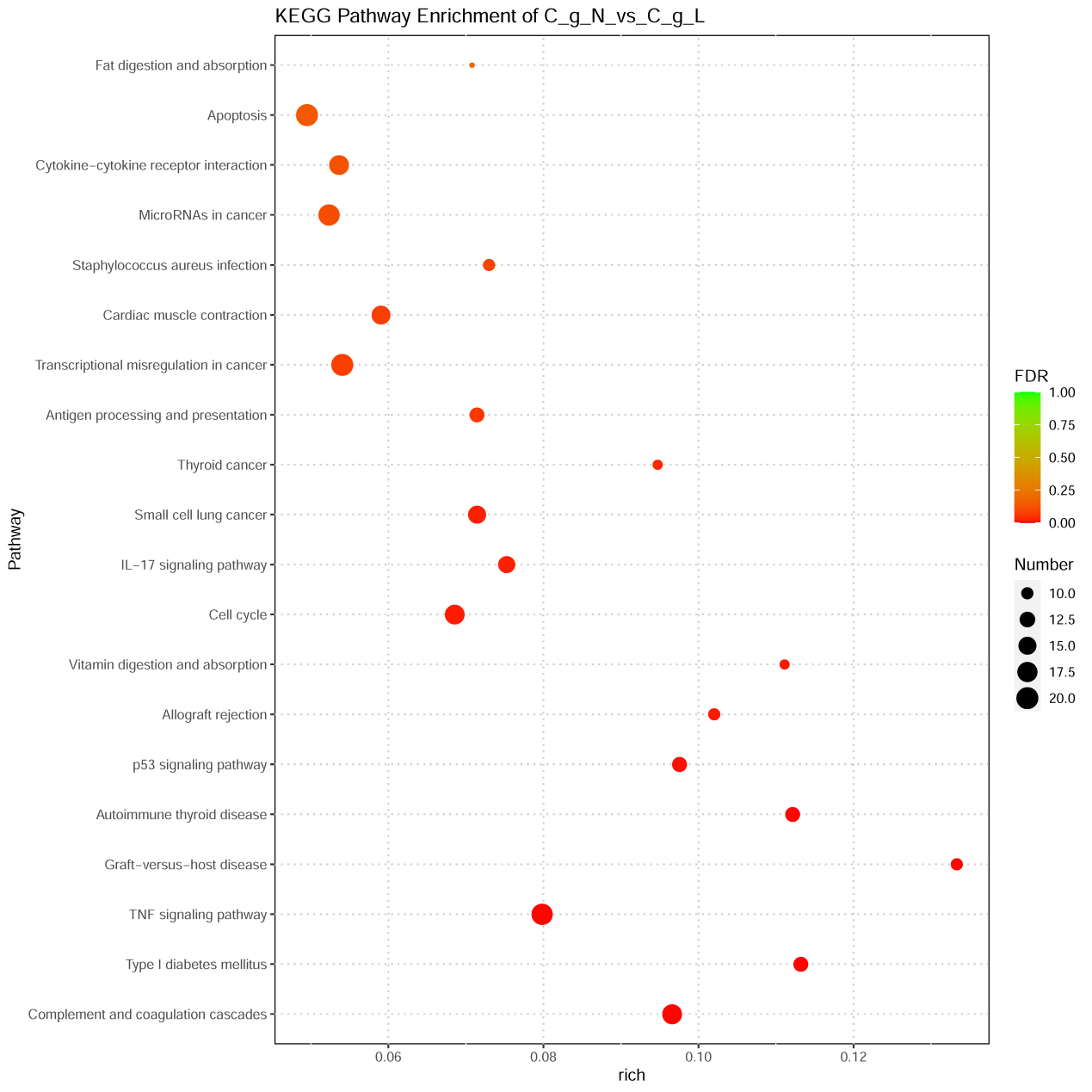


Fig. S4 The top 20 KEEG pathway enrichment of C_g_L vs C_g_N (Treat vs Control)

Note: C_g_N represents brain tissues at 22 ℃, C_g_L represents brain tissues at 4 ℃. Rich factor: [the number of differential genes enriched in the pathway]/[the number of differential genes annotated], the enrichment degree increases with the increase of rich. The general value range of FDR is 0 - 1, the enrichment degree increases with the decrease of FDR. Number represents the number of genes enriched in the pathway.


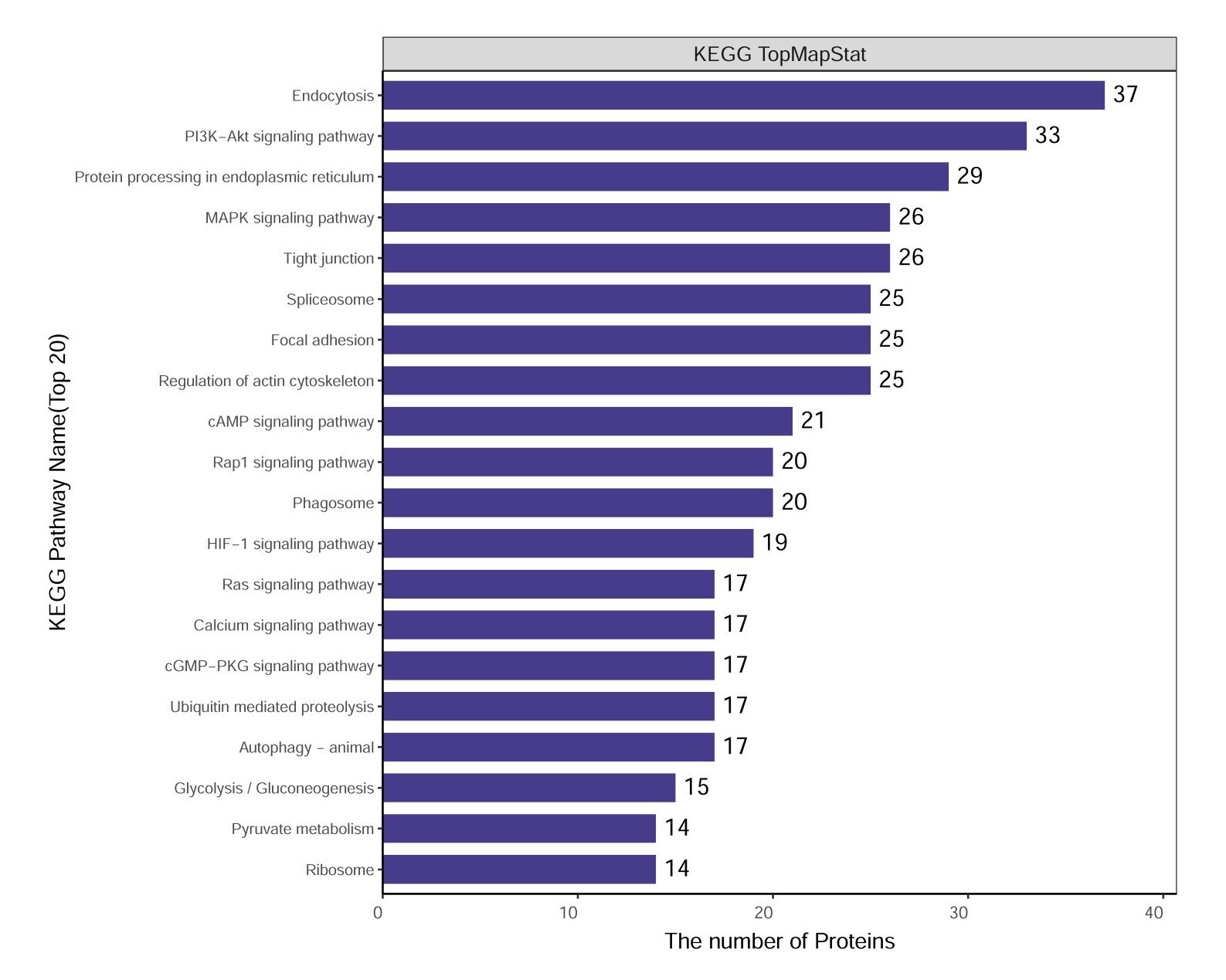


Fig. S5 The enrichment maps of the top 20 pathways of differentially expressed proteins of C_b_H vs C_b_N (Treat vs Control)

Note: C_b_N represents brain tissues at 22 ℃, C_b_H represents brain tissues at 30 ℃.


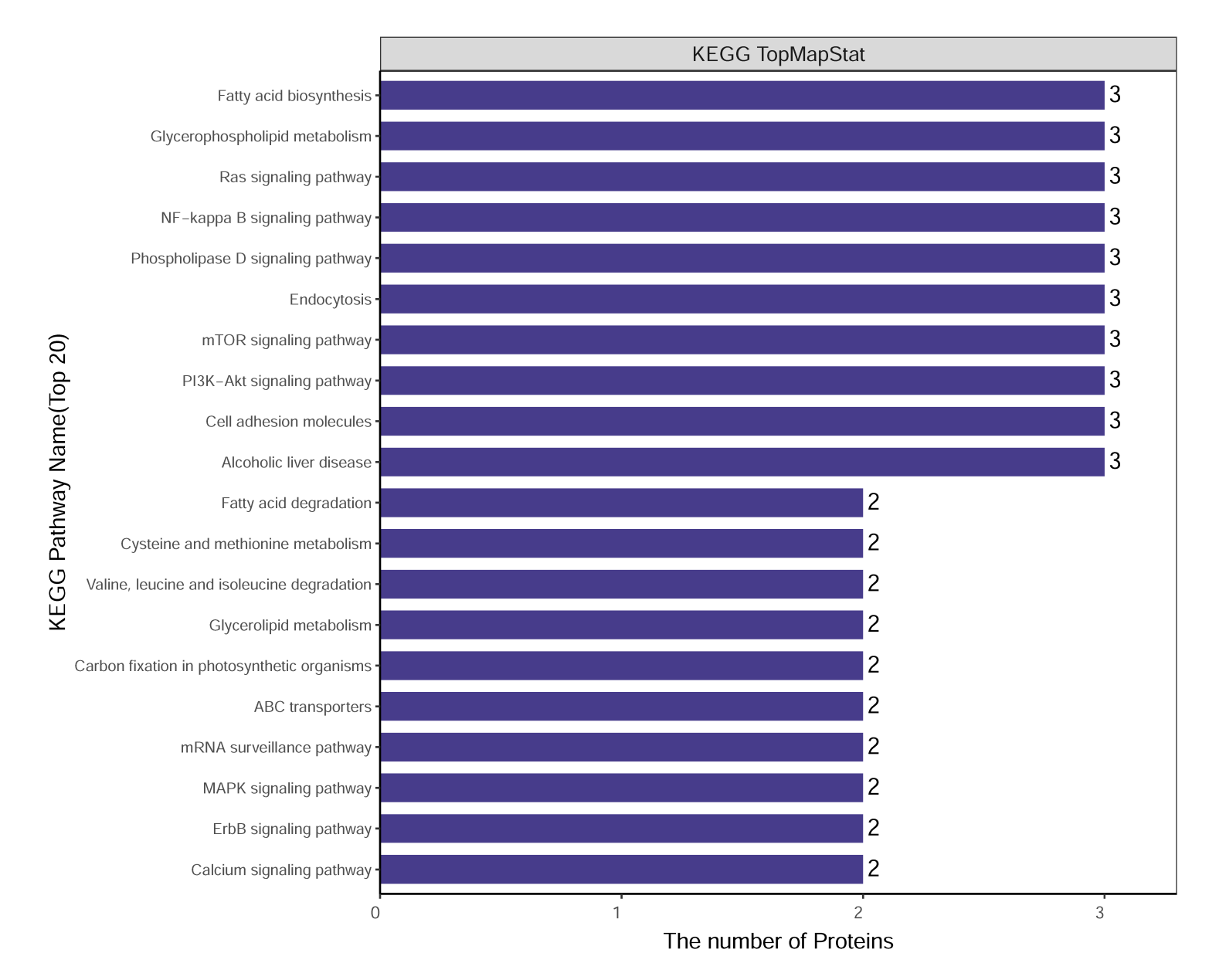


Fig. S6 The enrichment maps of the top 20 pathways of differentially expressed proteins of C_b_L vs C_b_N (Treat vs Control)

Note: C_b_N represents brain tissues at 22 ℃, C_b_L represents brain tissues at 4 ℃.


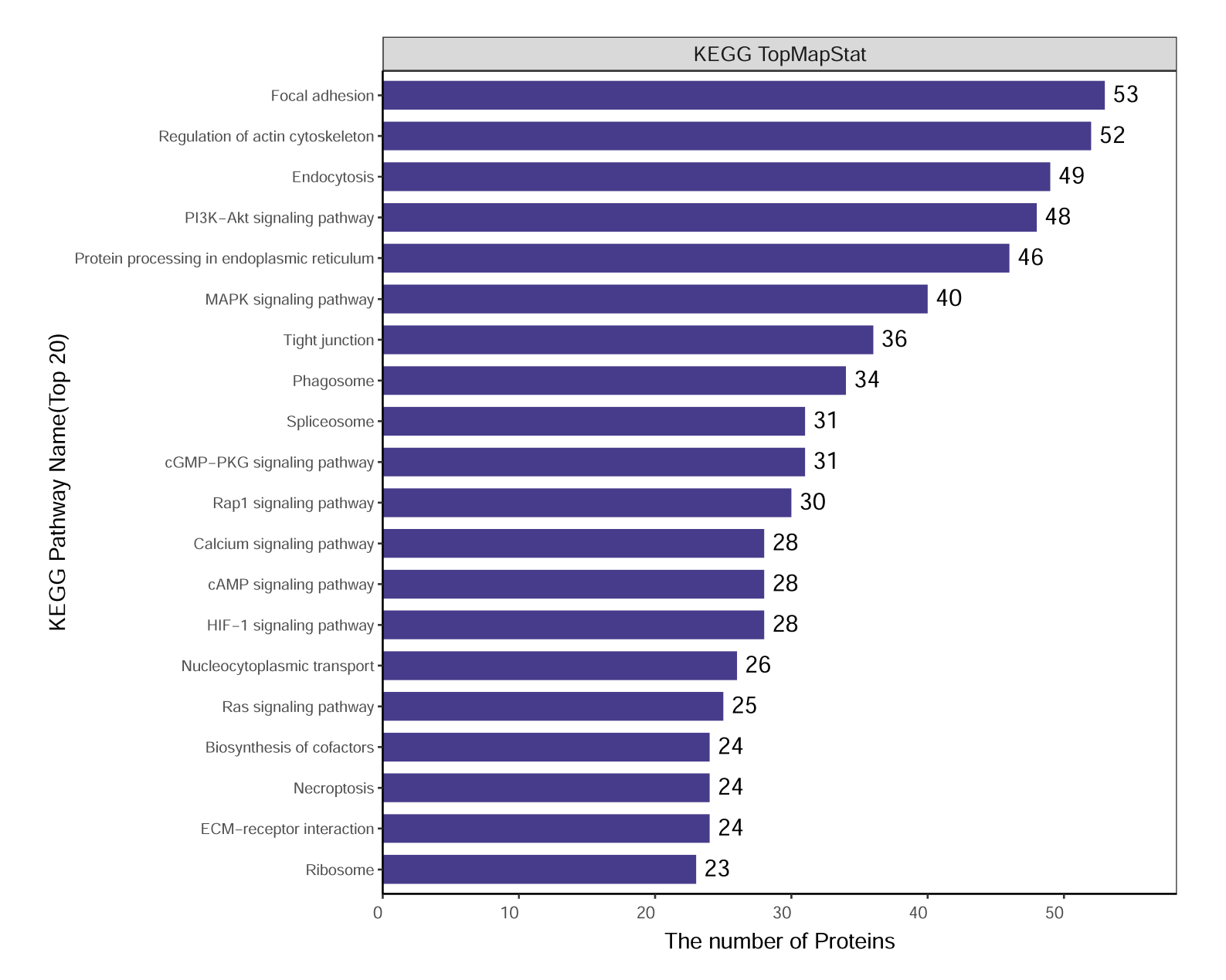


Fig. S7 The enrichment maps of the top 20 pathways of differentially expressed proteins of C_g_H vs C_g_N (Treat vs Control)

Note: C_g_N represents brain tissues at 22 ℃, C_g_H represents brain tissues at 30 ℃.


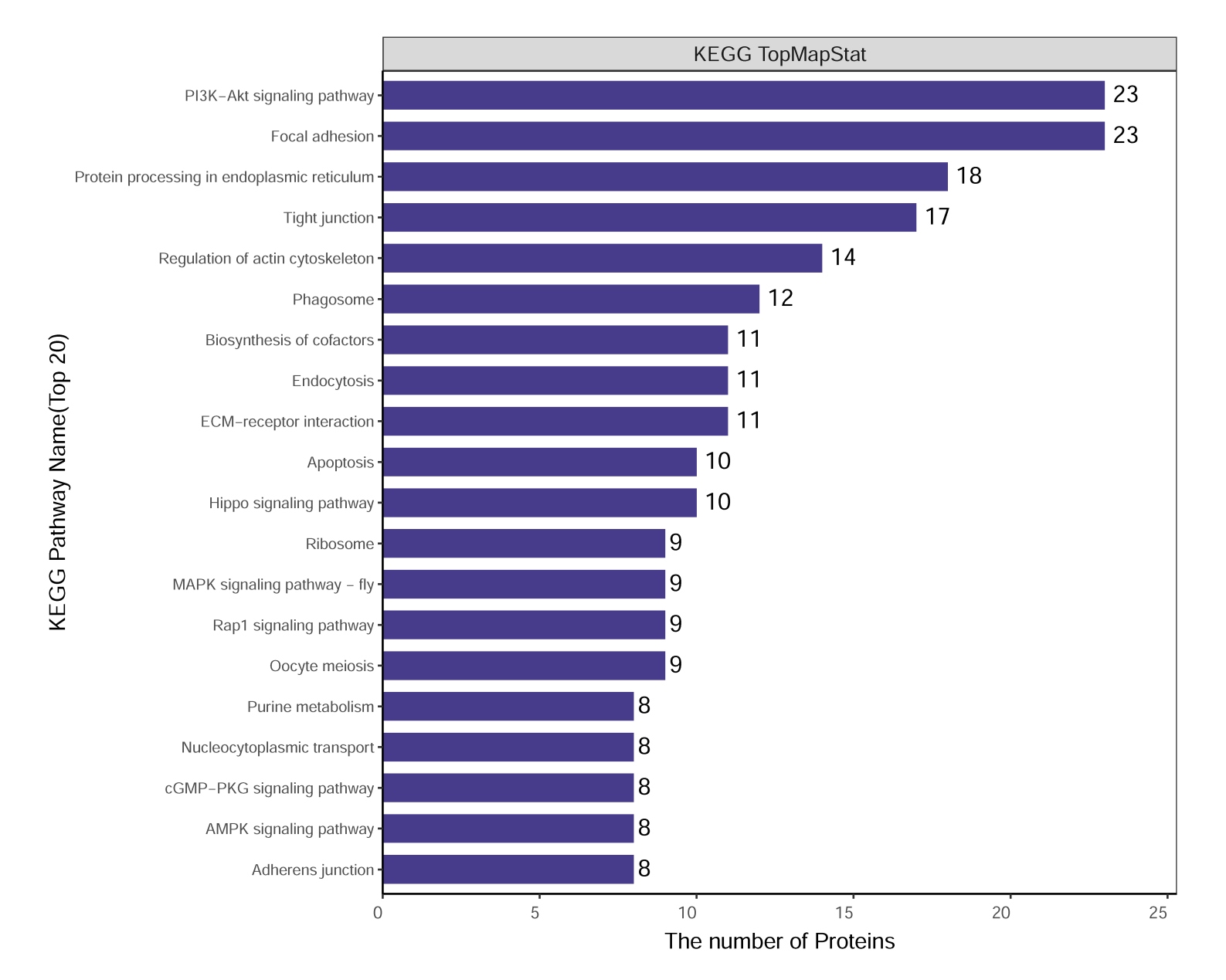


Fig. S8 The enrichment maps of the top 20 pathways of differentially expressed proteins of C_g_L vs C_g_N (Treat vs Control)

Note: C_g_N represents brain tissues at 22 ℃, C_g_L represents brain tissues at 4 ℃.


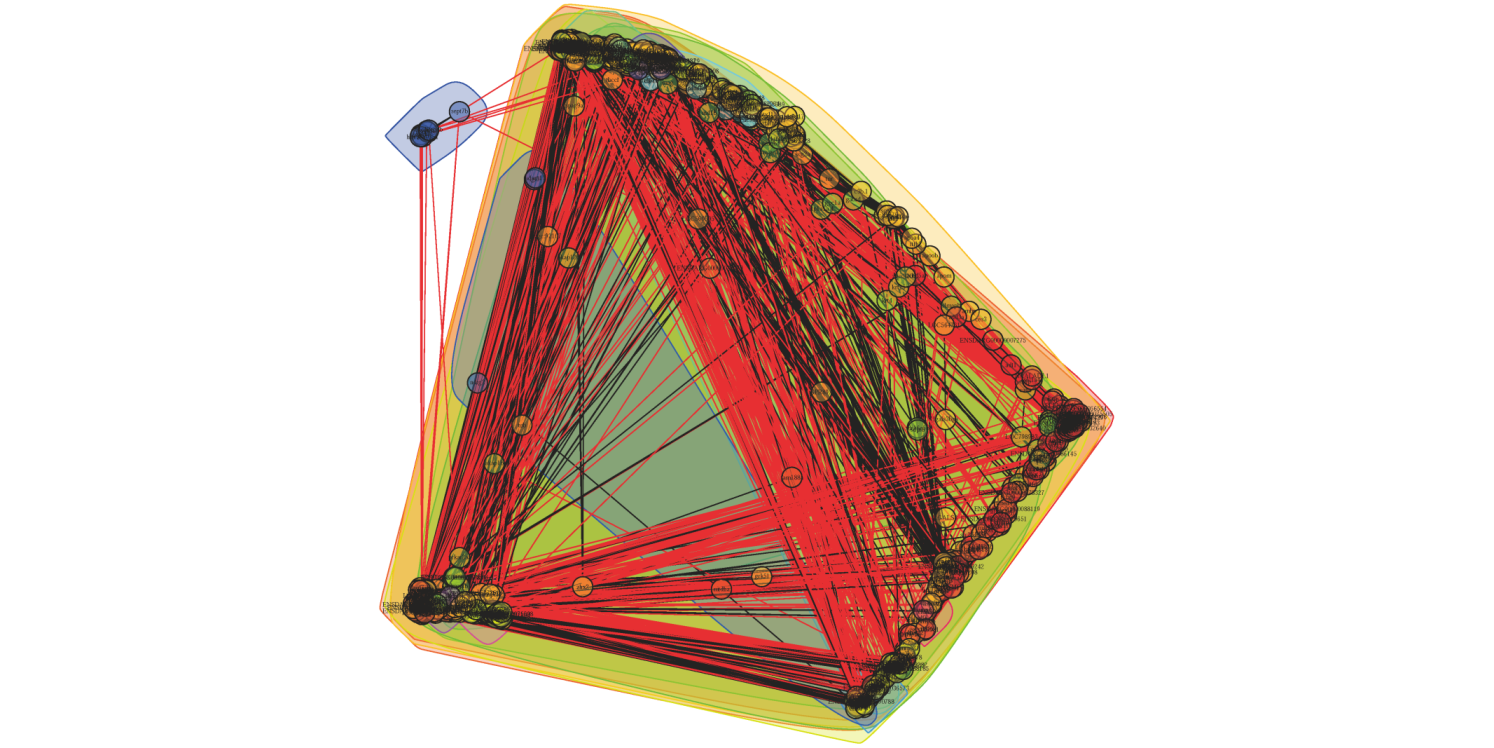


Fig. S9 The differentially expressed protein interaction network analysis of C_b_H vs C_b_N (Treat vs Control)

Note: C_b_N represents brain tissues at 22 ℃, C_b_H represents brain tissues at 30 ℃.


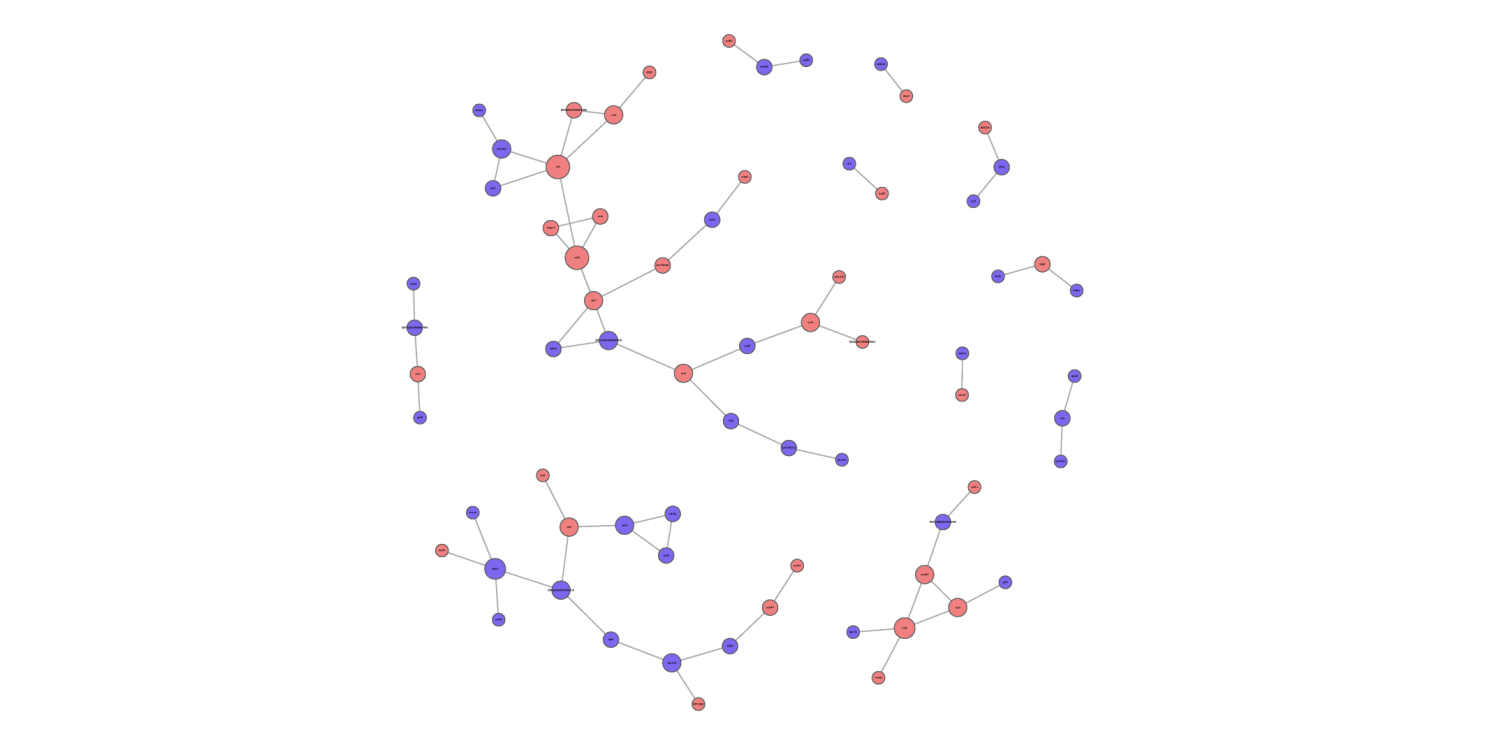


Fig. S10 The differentially expressed protein interaction network analysis of C_b_L vs C_b_N (Treat vs Control)

Note: C_b_N represents brain tissues at 22 ℃, C_b_L represents brain tissues at 4 ℃.


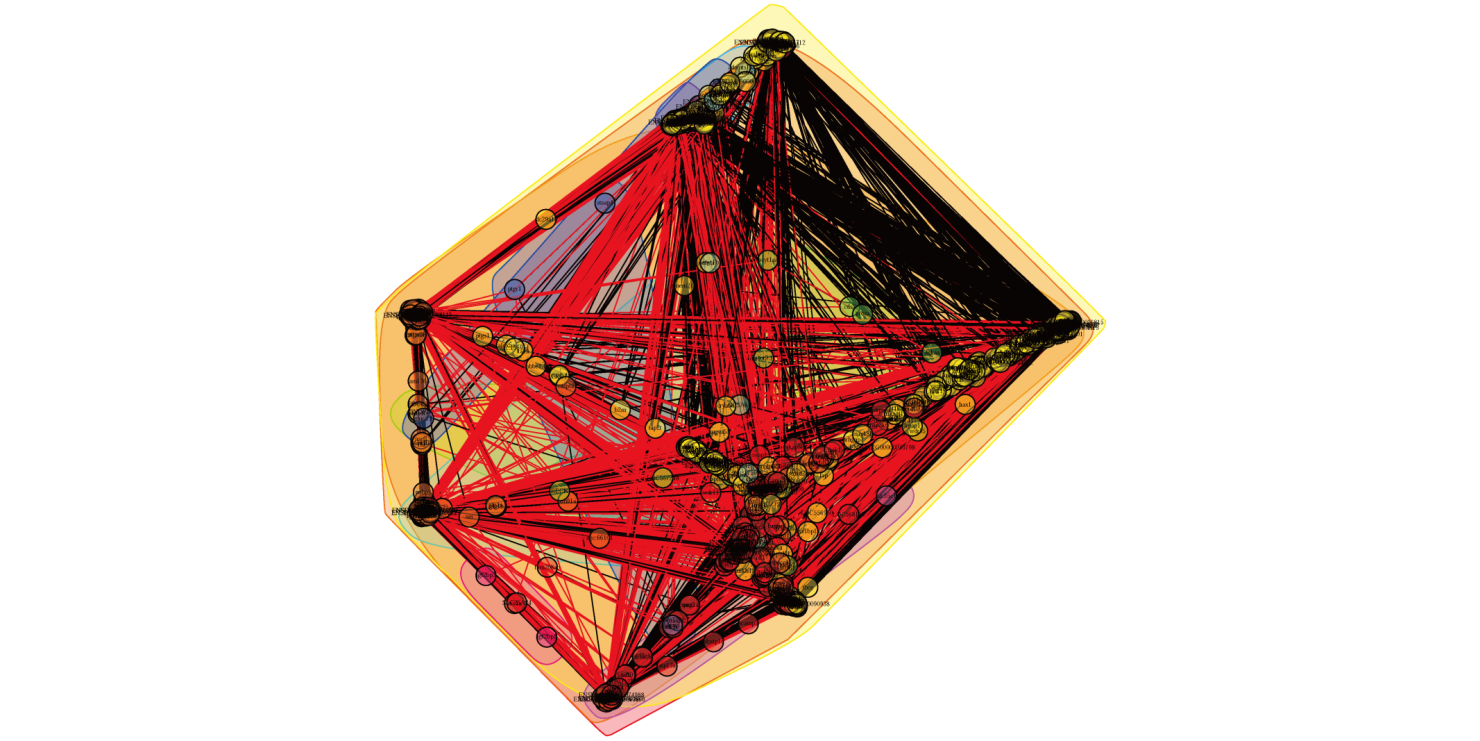


Fig. S11 The differentially expressed protein interaction network analysis of C_g_H vs C_g_N (Treat vs Control)

Note: C_g_N represents brain tissues at 22 ℃, C_g_H represents brain tissues at 30 ℃.


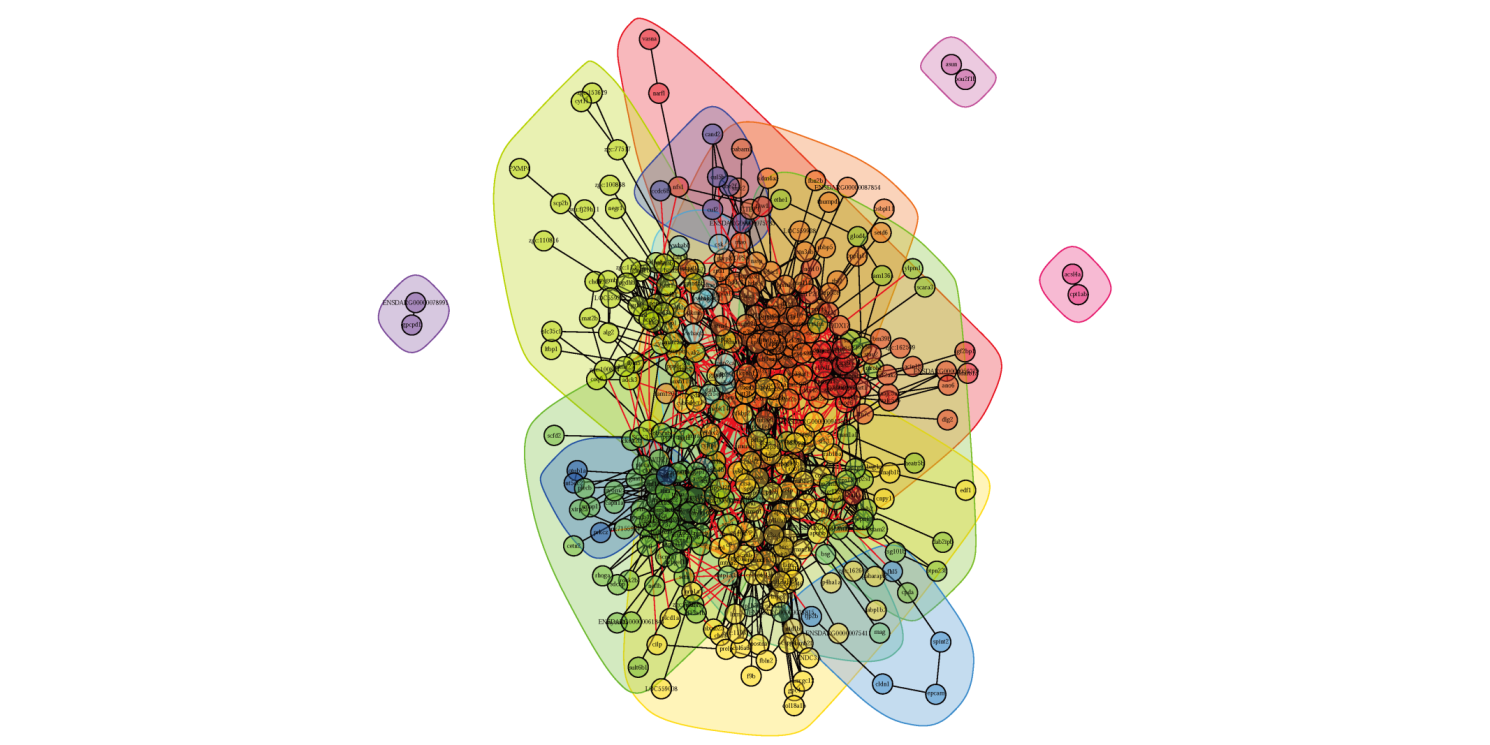


Fig. S12 The differentially expressed protein interaction network analysis of C_g_L vs C_g_N (Treat vs Control)

Note: C_g_N represents brain tissues at 22 ℃, C_g_L represents brain tissues at 4 ℃.
